# Quantitative description of the interactions among kinase cascades underlying long-term plasticity of *Aplysia* sensory neurons

**DOI:** 10.1038/s41598-021-94393-0

**Published:** 2021-07-22

**Authors:** Yili Zhang, Paul D. Smolen, Leonard J. Cleary, John H. Byrne

**Affiliations:** Department of Neurobiology and Anatomy, W.M. Keck Center for the Neurobiology of Learning and Memory, McGovern Medical School, The University of Texas Health Center At Houston, 6431 Fannin Street, Suite MSB 7.046, Houston, TX 77030 USA

**Keywords:** Computational neuroscience, Learning and memory

## Abstract

Kinases play critical roles in synaptic and neuronal changes involved in the formation of memory. However, significant gaps exist in the understanding of how interactions among kinase pathways contribute to the mechanistically distinct temporal domains of memory ranging from short-term memory to long-term memory (LTM). Activation of protein kinase A (PKA) and mitogen-activated protein kinase (MAPK)—ribosomal S6 kinase (RSK) pathways are critical for long-term enhancement of neuronal excitability (LTEE) and long-term synaptic facilitation (LTF), essential processes in memory formation. This study provides new insights into how these pathways contribute to the temporal domains of memory, using empirical and computational approaches. Empirical studies of *Aplysia* sensory neurons identified a positive feedforward loop in which the PKA and ERK pathways converge to regulate RSK, and a negative feedback loop in which p38 MAPK inhibits the activation of ERK and RSK. A computational model incorporated these findings to simulate the dynamics of kinase activity produced by different stimulus protocols and predict the critical roles of kinase interactions in the dynamics of these pathways. These findings may provide insights into the mechanisms underlying aberrant synaptic plasticity observed in genetic disorders such as RASopathies and Coffin-Lowry syndrome.

## Introduction

Extensive research has delineated ways in which kinases and growth factors play critical roles in synaptic and neuronal changes necessary for the formation of long-term memory (LTM). However, many details of the dynamics of these cascades, their cross talk and feedback interactions, remain to be elucidated. These details are necessary to understand the specific contributions of these processes to the distinct temporal domains of memory, which range from minutes to days to a lifetime. PKA and MAPK pathways are believed to be essential for synaptic plasticity and changes in intrinsic neuronal excitability, both essential for the formation of LTM^1–9^. Current data indicate a high degree of complexity in the dynamics of these pathways^10^ including biphasic regulation of kinases^11^. In addition, it is clear that feedback loops involving extracellular signaling molecules, such as growth factors, are important for long-term changes in excitability and synaptic plasticity, and thus for formation of LTM^12–18^. Therefore, understanding the induction of these long-term changes will require detailed empirical investigation, complemented by mathematical modeling.

The *Aplysia* sensorimotor synapse provides a useful system to elucidate these interactions and feedback loops. PKA and the MAPK isoform extracellular signal-regulated kinase (ERK) converge to regulate genes (e.g., *c/ebp*) critical for serotonin (5-HT)-induced long-term synaptic facilitation (LTF), long-term enhancement of intrinsic excitability (LTEE) in sensory neurons (SNs), and LTM^8,9,19^. LTF and LTEE can be studied in vitro using a Standard protocol consisting of five pulses of 5-HT with an interstimulus interval (ISI) of 20 min^13,20–22^, or using an Enhanced protocol of five pulses of 5-HT with irregular ISIs^23^. LTEE can be produced in isolated SNs in the absence of postsynaptic targets^22–24^, indicating that molecular cascades intrinsic to SNs are sufficient to induce that change. Besides the five-pulse 5-HT protocols, LTM can also be produced by two pulses of 5-HT with an ISI of 45 min^25^.

Insights into the dynamics of signaling cascades underlying LTF have been obtained with a single 5-min treatment of 5-HT. In SNs, activation of PKA is immediate but transient, returning to the basal activity about 5 min after the end of the 5-HT pulse^26^, whereas activation of ERK is delayed until ~ 45 min after treatment and persists for ~ 15 min^25^. These temporal differences generate a requirement for multiple training pulses to induce LTF^20,21,27, 28^. The total duration of spaced pulses must be sufficient to allow overlap of PKA and ERK activation^28^. Recent studies have revealed unappreciated dynamics and roles of other kinases activated by 5-HT, including p38 MAPK and p90 ribosomal S6 kinase (RSK)^11,23^. Activation of p38 MAPK suppresses LTF in *Aplysia*, and its activity is reduced by 5-HT^29–31^. Recent results demonstrate a biphasic time course of p38 MAPK activation^11^. A 5-min application of 5-HT inhibits p38 MAPK^11,29,30^, followed by activation 45 min later^11^. This activation is due to MEK, a kinase upstream of ERK. In addition, inhibition of p38 MAPK prolongs ERK activation. These data suggest reciprocal interaction between ERK and p38 MAPK. RSK in *Aplysia* is activated by the MEK/ERK pathway and RSK activity contributes to 5-HT-induced phosphorylation of the transcription activator cAMP response element binding protein 1 (CREB1), as well as LTF^23^.

These new findings raise the possibility that other interactions are present and contribute to the dynamics of kinases and induction and consolidation of LTF and LTEE. To address this possibility, we first examined the dynamics of PKA and MAPK pathways in *Aplysia* SNs after 5-HT in the presence and absence of kinase inhibitors. Two new pathways were identified: PKA-dependent, but ERK-independent, regulation of RSK activity, and RSK-dependent activation of p38 MAPK. These data and other recent results were used to extend our previous computational model of multiple molecular cascades underlying LTF and LTEE. The model includes the dynamics of two growth factors, *Aplysia* neurotrophin (NT) and transforming growth factor-β (TGF-β), both of which play critical roles in MAPK activation^13,17,18^. The model simulates kinase activation after different training regimens, maintains the predictive ability of the previous model^28^, and prompts new predictions validated by subsequent experiments. To date this model represents the most detailed quantitative description of the complex interactions among the kinase pathways and growth factor cascades necessary for LTF and LTEE in SNs.

## Results

An electrical shock delivered to the tail, shock of the nerve innervating the tail, or a direct exposure of SNs to 5-HT, leads to activation of ERK and p38 MAPK^11,25,32^, but there is a lack of understanding of the dynamics of this activation, and of the dynamics of activation of PKA and RSK, which interact with these MAPKs. Here, immunofluorescence analysis was used to measure these dynamics.

### One pulse of 5-HT induces two waves of increased phosphorylated RSK

**Figure 1:**
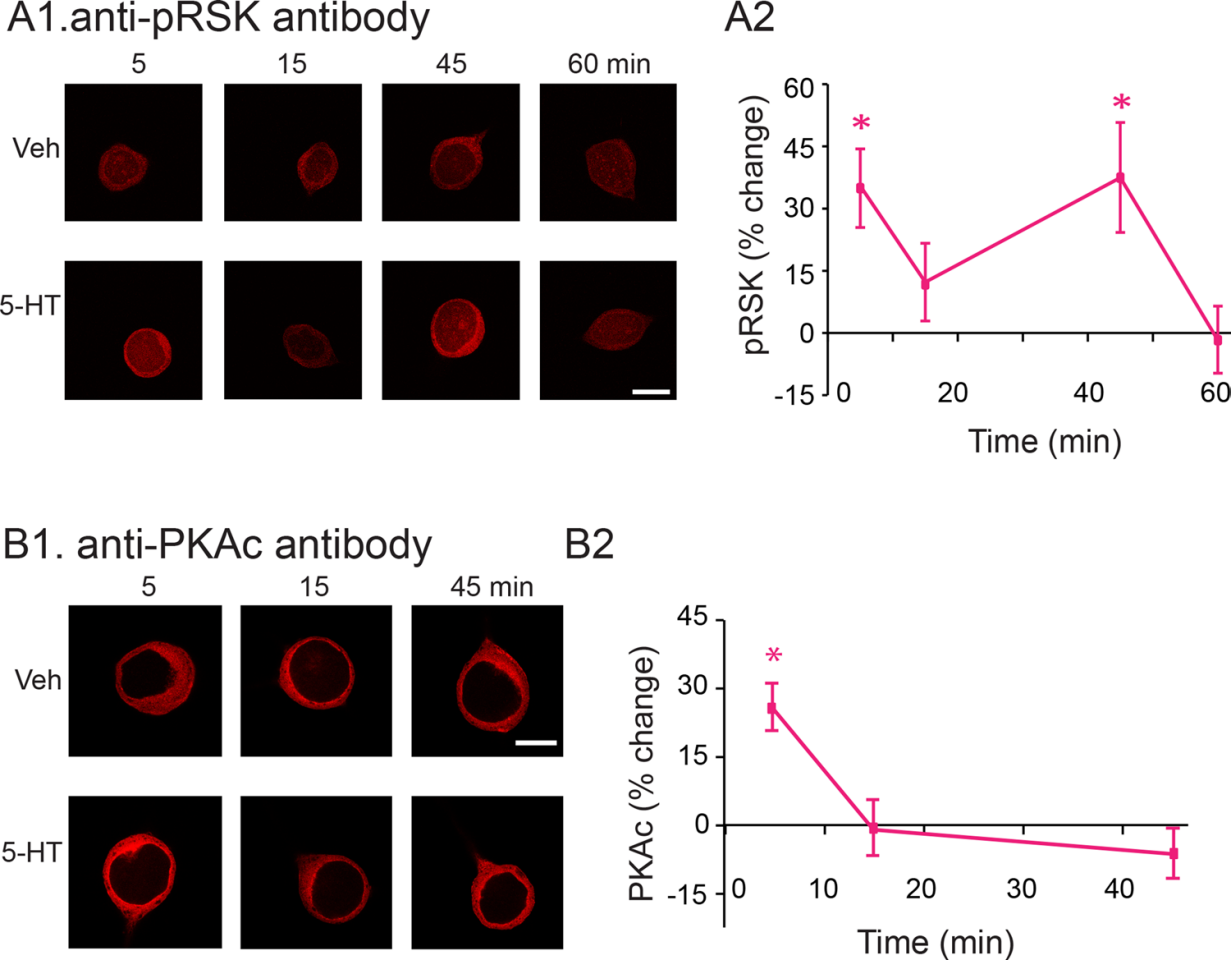
Dynamic regulation of pRSK (**A**) and PKA catalytic subunits (PKAc) (**B**) by a brief (5 min) pulse of 50 μM 5-HT. A1, Representative confocal images of pRSK immunofluorescence in SNs at different times post-onset of 5-HT. A2, Summary data. The percent change was calculated as the change of pRSK level after 5-HT compared to time-matched control levels. pRSK increased immediately after 5-HT (n = 8), returned to basal level at 15 min (n = 9), followed by a delayed increase at about 45 min (n = 13), and then a return to basal level at 60 min (n = 8). B1, Representative confocal images of PKAc at different times post-onset of 5-HT. B2, Summary data. The percent change was calculated as the change of PKAc level after 5-HT compared to time-matched control levels. PKAc increased immediately after 5-HT (n = 10), returned to basal level at 15 min (n = 7), and remained at basal level at 45 min (n = 8). Data are represented as mean ± SEM. All scale bars are 40 μm. *p < 0.05.

The increase of active, phosphorylated RSK (pRSK) 1 h after five pulses of 5-HT is blocked by a MEK inhibitor^23^, suggesting RSK is downstream of MEK and ERK. Therefore, we expected the dynamics of pRSK after 5-HT treatment to be similar to those of phosphorylated ERK (pERK). We measured levels of pRSK at 5, 15, 45 and 60 min post-onset of a 5-min pulse of 5-HT. 5-HT induced two waves of increase in pRSK (Fig. 1A). RSK phosphorylation increased immediately after treatment (33.3 ± 9.5%), and returned towards basal at 15 min (13.8 ± 9.1%), followed by a delayed increase at about 45 min (36.3 ± 13.8%), and then a return to basal level at 60 min (− 1.66 ± 6.8%). Statistical analyses (WSRT using Bonferroni corrections, Methods) revealed that the increases at 5 min (immediately after the 5-HT pulse ended) and 45 min post-onset of 5-HT were significant compared to Veh controls (at 5 min, Z = 2.521, *P* = 0.032; at 45 min, Z = 2.481, *P* = 0.04), whereas those at 15 min (Z = 0.77, *P* = 1.984), and 60 min (Z = − 0.56, *P* = 2.564) were not. The early increase in pRSK was surprising given that pERK, the presumed activator of RSK, does not increase significantly until about 45 min post-onset^25^. Thus, this early RSK activation is likely due to an ERK-independent pathway.

### One pulse of 5-HT only transiently increases the level of the catalytic subunit of PKA

One pulse of 5-HT induces an immediate increase of PKA activity in *Aplysia*, which returns to basal activity within 15 min^26^, but later time points were not previously examined. To be consistent with other experiments^11,25^, we attempted to confirm and extend those results. Because the level of catalytic subunit of PKA is an indicator of PKA activity^18^, we used an antibody directed against this subunit (anti-PKAc, Abcam) and verified antibody specificity by observing an increase of PKAc level upon 5-HT activation of the cAMP pathway (Fig. S1)^23,33,34^. We then measured PKAc at 5, 15, and 45 min after the end of the 5-HT pulse (Fig. 1B). PKAc increased immediately after treatment (25.7 ± 5.1%), returned towards basal level at 15 min (− 0.5 ± 5.5%), and remained basal at 45 min (− 5.4 ± 4.9%). Statistical analyses revealed that the increase at 5 min (i.e., immediately after the end of 5-HT) was significant compared to Veh control (at 5 min, t_9_ = − 5.400, *P* = 0.003), whereas any changes at 15 min (t_6_ = 0.319, *P* = 2.28), and 45 min (t_7_ = 1.142, *P* = 0.873) were not. These results suggest that one pulse of 5-HT only induced a transient increase in the level of PKAc, a fundamentally different time course from pRSK (Fig. 1A), pERK^25^ and phosphorylated p38 MAPK (p-p38 MAPK)^11^. The increase of PKAc is dependent on the cAMP pathway (Fig. S1). cAMP induces a separation of catalytic and regulatory subunits^6^. However, the increase of PKAc detected by the anti-PKAc antibody used in this study could also be caused by rapid increase in synthesis or decrease in degradation of PKAc.

### PKA mediates the delayed increase of pERK

**Figure 2:**
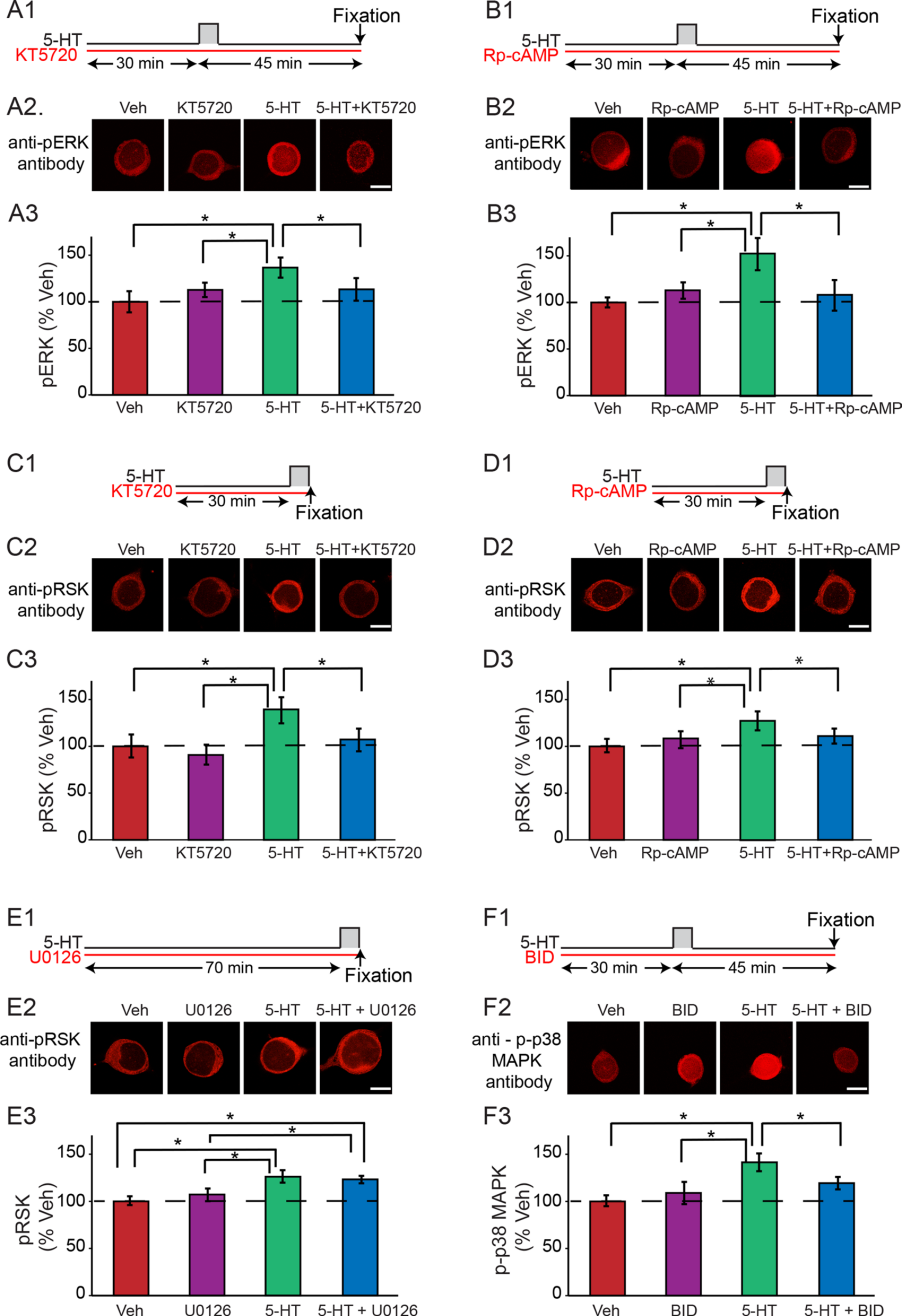
The interactions between PKA and ERK (**A**,**B**), PKA and RSK (**C**,**D**), RSK and p38 MAPK (**E**). A1, Protocol for applying the PKA inhibitor KT5720 with 5 min 5-HT. A2, Representative confocal images of pERK in SNs at 45 min post-onset of 5-HT, in the absence or presence of KT5720. A3, Summary data. KT5720 significantly decreased pERK induced by 5-HT (n = 10). B1, Protocol for applying the PKA inhibitor Rp-cAMP with 5-HT. B2, Representative confocal images of pERK at 45 min post-onset, in the absence or presence of Rp-cAMP. B3, Summary data. Rp-cAMP significantly decreased pERK induced by 5-HT (n = 6). C1, Protocol. C2, Representative confocal images of pRSK immediately after 5-HT, in the absence or presence of KT5720. C3, Summary data. KT5720 significantly decreased pRSK induced by 5-HT (n = 7). D1, Protocol. D2, Representative confocal images of pRSK immediately after 5-HT, in the absence or presence of Rp-cAMP. D3, Summary data. Rp-cAMP significantly decreased pRSK induced by 5-HT (n = 9). E1, Protocol for applying the MEK inhibitor U0126 with 5-HT. E2, Representative confocal images of pRSK immediately after 5-HT, in the absence or presence of U0126. E3, Summary data. U0126 did not significantly attenuate the increase of pRSK immediately after 5-HT (n = 6). F1, Protocol for applying the RSK inhibitor BI-D1870 (BID) with 5-HT. F2, Representative confocal images of p-p38 MAPK at 45 min post-onset of 5-HT, in the absence or presence of BID. F3, Summary data. BID attenuated the induction of p-p38 MAPK 45 min post-onset of 5-HT (n = 9). Data are represented as mean ± SEM. All scale bars are 40 μm. *p < 0.05.

**Figure 3:**
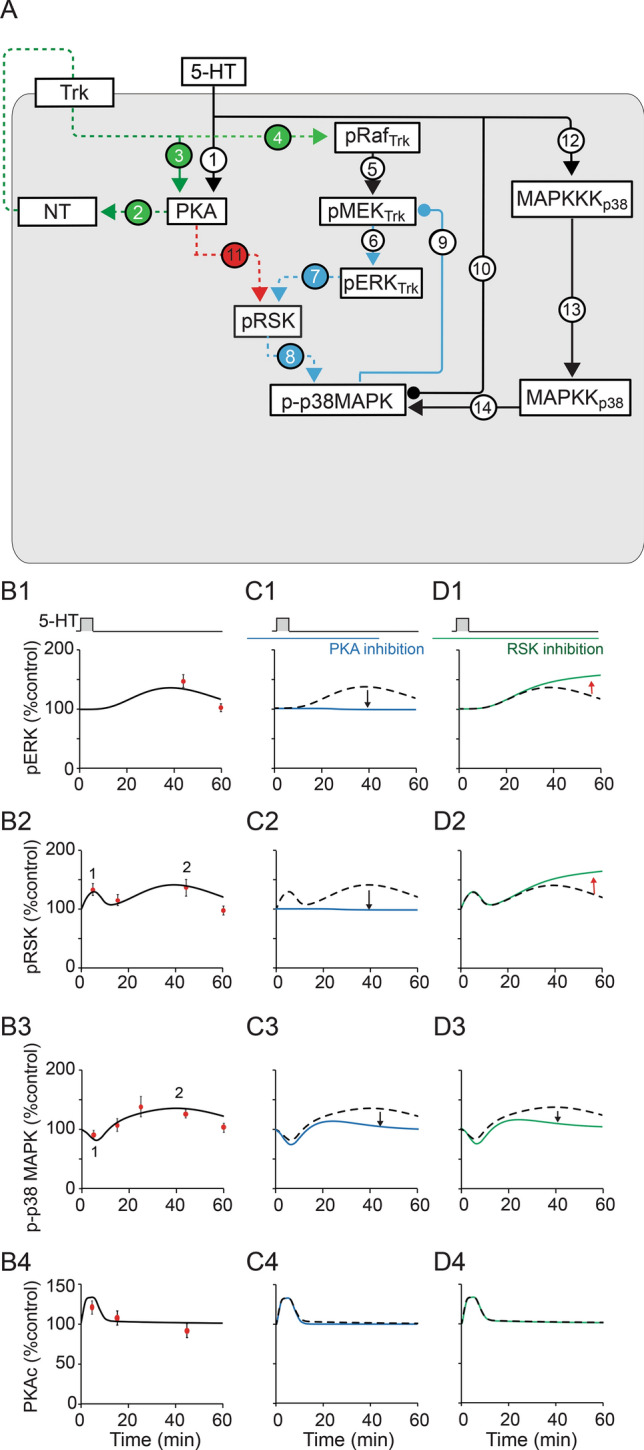
(**A**) Schematic model of PKA and MAPK signaling pathways. 5-HT regulates the PKA and MAPK cascades via multiple pathways. Red, blue, green dashed lines represent newly added pathways. Blue denotes the ERK/RSK/p38 MAPK feedback loop, green denotes the NT-dependent pathways. Each number represents a signaling pathway (not equation numbers). Arrowheads indicate activation, circular ends indicate repression. (**B**–**D**) Simulated dynamics of kinases after one pulse of 5-HT. (**B**) Control simulations (black solid curves). Empirical data points (red circles) are from this study (PKAc and pRSK) and from Zhang et al.^11^ (pERK and p-p38 MAPK). Numbers “1”, “2” in B2 represent two waves of increases in pRSK. The “1” in B3 represents the transient decrease of p-p38 MAPK. The “2” in B3 represents the delayed increase of p-p38 MAPK. (**C**) Dynamics of kinases with a simulated inhibition of downstream effects of PKA, ended 45 min post-onset of 5-HT. The slow increases of pERK (C1), pRSK (C2) and p-p38 MAPK (C3) were blocked. Blue curves are simulations with inhibitors, black dashed curves are control simulation. This inhibition suppressed the delayed activation of pERK as observed empirically in Fig. 2A, and pRSK and p-p38 MAPK subsequently decreased (black arrows). Control simulation curves added in this and following panels and figures, are for the convenience of comparison with the curves in the presence of inhibitors. Dashed curves are used to make overlapped curves visible. (**D**) Dynamics of kinases with simulated inhibition of RSK ending 60 min post-onset of 5-HT. The delayed increase of p-p38 MAPK (D3) was blocked by RSK inhibition, but pERK (D1) and pRSK (D2) remained elevated for 60 min. Green curves are simulations with inhibitors, black dashed curves are control simulation. This RSK inhibition suppressed the delayed activation of p-p38 MAPK (D3, black arrow) as observed empirically (Fig. 2F). Reduced p-p38 MAPK activity disinhibited MEK. The disinhibition led to a sustained increase of pERK and pRSK at 1 h (D1-2, red arrows).

A single pulse of 5-HT induces a delayed increase in ERK at 45 min^25^, possibly via growth factor signaling activated by PKA^3^. This delayed increase of MAPK activity is blocked by a human recombinant tropomyosin receptor kinase B (TrkB) antagonist^17^. The molecular pathway(s) in *Aplysia* affected by this TrkB inhibitor remain unclear^35–37^. However, *Aplysia* neurotrophin (NT) is an ortholog of vertebrate neurotrophin. Its *Aplysia* Trk receptor can specifically bind the vertebrate TrkB ligand BDNF^38^. Release of NT depends on PKA^38^. Moreover, NT-Trk signaling is known to increase pERK in PC12 cells and mediate 5-HT-induced LTF in *Aplysia*^18,38^. Therefore, we hypothesized that the Trk-dependent ERK activation peaking ~ 45 min after the onset of 5-HT was due to PKA activation, leading to release of NT and activation of Trk receptors. Activation of Trk would lead to ERK activation (Fig. 3A, pathway 1 → 2 → 4 → 5 → 6). To test this hypothesis, levels of pERK were examined in the presence of the PKA inhibitor KT5720 (Fig. 2A1) (Methods). Example responses and summary data are illustrated in Figs. 2A2–A3. 5-HT alone increased pERK by 36.7 ± 10.8%, whereas the increase was reduced (13.3 ± 12.1%) in the presence of KT5720. Pairwise comparisons (Student-Newman–Keuls, SNK) following a repeated measures one-way ANOVA (RM ANOVA) revealed that the 5-HT alone group was significantly different from the other groups (Methods, Table S1). These results were replicated with a second PKA inhibitor, Rp-cAMP, which also significantly reduced pERK ~ 45 min post-onset of 5-HT (Fig. 2B, Table S1). Because one pulse of 5-HT transiently increased PKA catalytic subunits (Fig. 1B), the results of Fig. 2A,B support the hypothesis that PKA induces the delayed increase of pERK, via a slow pathway such as the PKA-NT-ERK pathway (Fig. 3A, pathway 1 → 2 → 4 → 5 → 6).

### PKA mediates the initial increase of pRSK immediately after 5-HT

The initial increase in pRSK in response to one pulse of 5-HT (Fig. 1) was unexpected given that its presumed activator, pERK, is not elevated at that time^25^. However, data suggest that RSK can be regulated by PKA via an ERK-independent pathway. In mouse lung fibroblasts, silencing expression of the regulatory PKARIα subunit increased activation of RSK, in the absence of changes in ERK activity^39^. Therefore, we quantified pRSK immediately after one 5-min pulse of 5-HT in the absence or presence of the PKA inhibitor KT5720 (Fig. 2C1). Example responses and summary data are illustrated in Figs. 2C2–C3. 5-HT alone led to a 42.0 ± 14.3% increase in pRSK. With KT5720, 5-HT led to only an 8.1 ± 11.9% increase in pRSK. Pairwise comparisons following a RM ANOVA revealed that the 5-HT alone group was significantly different from the other three groups (Table S1). These results were replicated with Rp-cAMP, which also significantly reduced the increase of pRSK immediately after 5-HT (Fig. 2D, Table S1). In addition, we also measured pRSK immediately after one 5-min pulse of 5-HT in the absence vs*.* presence of U0126, the inhibitor of MEK, the kinase that activates ERK (Fig. 2E1). Example responses and summary data are illustrated in Figs. 2E2–E3. U0126 did not significantly change the level of pRSK immediately after 5-HT (n = 6). 5-HT alone led to a 25.0 ± 4.9% increase in pRSK. With U0126, 5-HT led to a 20.9 ± 2.1% increase in pRSK. Pairwise comparisons following a RM ANOVA revealed that both the 5-HT alone group and the 5-HT plus U0126 group were significantly different from the Veh and U0126 alone groups, but there was no significant difference between these two groups (Table S1). These data indicate that PKA, not ERK, mediates the initial increase of pRSK (Fig. 3A, pathway 11), and that PKA can activate RSK via two pathways, one ERK-independent (Fig. 3A, pathway 11), and a second ERK-dependent (Fig. 3A, pathway 2 → 4 → 5 → 6), thus forming a positive PKA—RSK feedforward loop.

### RSK contributes to the delayed increase of p-p38 MAPK

One pulse of 5-HT induces a delayed increase of p-p38 MAPK approximately 45 min post-onset. This increase can be suppressed by the MEK inhibitor U0126^11^. This result does not distinguish between a direct action of MEK on p38 MAPK and an action on a kinase downstream of MEK, such as RSK. To investigate whether p38 MAPK is phosphorylated via the MEK/ERK/RSK pathway (Fig. 3A, pathway 5 → 6 → 7 → 8), we quantified p-p38 MAPK at 45 min post-onset of 5 min 5-HT, in the absence or presence of the RSK inhibitor BI-D1870 (Fig. 2F1). Example responses and summary data are illustrated in Figs. 2F2—2F3. 5-HT alone led to a 38.9 ± 9.1% increase in p-p38 MAPK at 45 min. In contrast, treatment with 5-HT plus BI-D1870 led to a 16.7 ± 5.7% increase in p-p38 MAPK. Because these results showed a non-normal distribution, Friedman repeated measures analysis of variance on ranks was used and demonstrated a significant overall effect of the treatments (Table S1). Pairwise comparisons (SNK) revealed that the 5-HT alone group was significantly different from the other three groups (Table S1). Therefore, RSK inhibition attenuated the delayed increase of p-p38 MAPK. Combined with previous results that MEK inhibition also reduced the delayed increase of p-p38 MAPK^11^, the results suggest that activation of p38 MAPK in *Aplysia* is regulated by the MEK/ERK/RSK pathway (Fig. 3A, pathway 6 → 7 → 8).

### Computational model of signaling pathways induced by 5-HT

#### Simulation of kinase dynamics after one pulse of 5-HT

We first developed a model of PKA and MAPK signaling cascades activated by one pulse of 5-HT (Fig. 3A, see also Methods) based on results from this and previous studies. In Fig. 3B, empirical results are given as red circles, simulated activities by black lines. One pulse of 5-HT induced one wave of increase in pERK via the PKA/NT/Trk pathway (Fig. 3A, pathway 1 → 2 → 4 → 5 → 6), but two waves of increase in pRSK (‘1’, ‘2’ in Fig. 3B2). The first wave was independent of the NT-ERK pathway (Fig. 3A, pathway 1 → 11) whereas the second was via the NT-ERK pathway (Fig. 3A, pathway 1 → 2 → 4 → 5 → 6 → 7). p-p38 MAPK initially decreased (‘1’ in Fig. 3B3), followed by an increase (‘2’ in Fig. 3B3). The initial decrease was independent of the ERK-RSK pathway (Fig. 3A, pathway 10), whereas the delayed increase was dependent on the NT-ERK-RSK pathway (Fig. 3A, pathway 1 → 2 → 4 → 5 → 6 → 7 → 8). The decay of pERK, pRSK and p-p38 MAPK to basal levels at 60 min is due to negative feedback from p38 MAPK (Fig. 3A, pathway 9).

Figure 3C,D illustrate simulated responses of kinases to the empirical treatments of Fig. 2. For Fig. 3C, pathways 2 and 11 (Fig. 3A) were blocked from 30 min prior to until 45 min post-onset of 5-HT to simulate an inhibition of downstream effects of PKA (same as the effect of KT5720). For Fig. 3D, pathway 8 in Fig. 3A was blocked from 30 min prior to until 60 min post-onset of 5 min 5-HT, to simulate the RSK inhibition. Both these simulations replicated the corresponding empirical results (Fig. 2A,F). Thus, the model of Fig. 3A simulates empirical data obtained with one pulse of 5-HT in the absence or presence of kinase inhibitors.

#### Simulated responses to two pulses of 5-HT using an extended model

**Figure 4:**
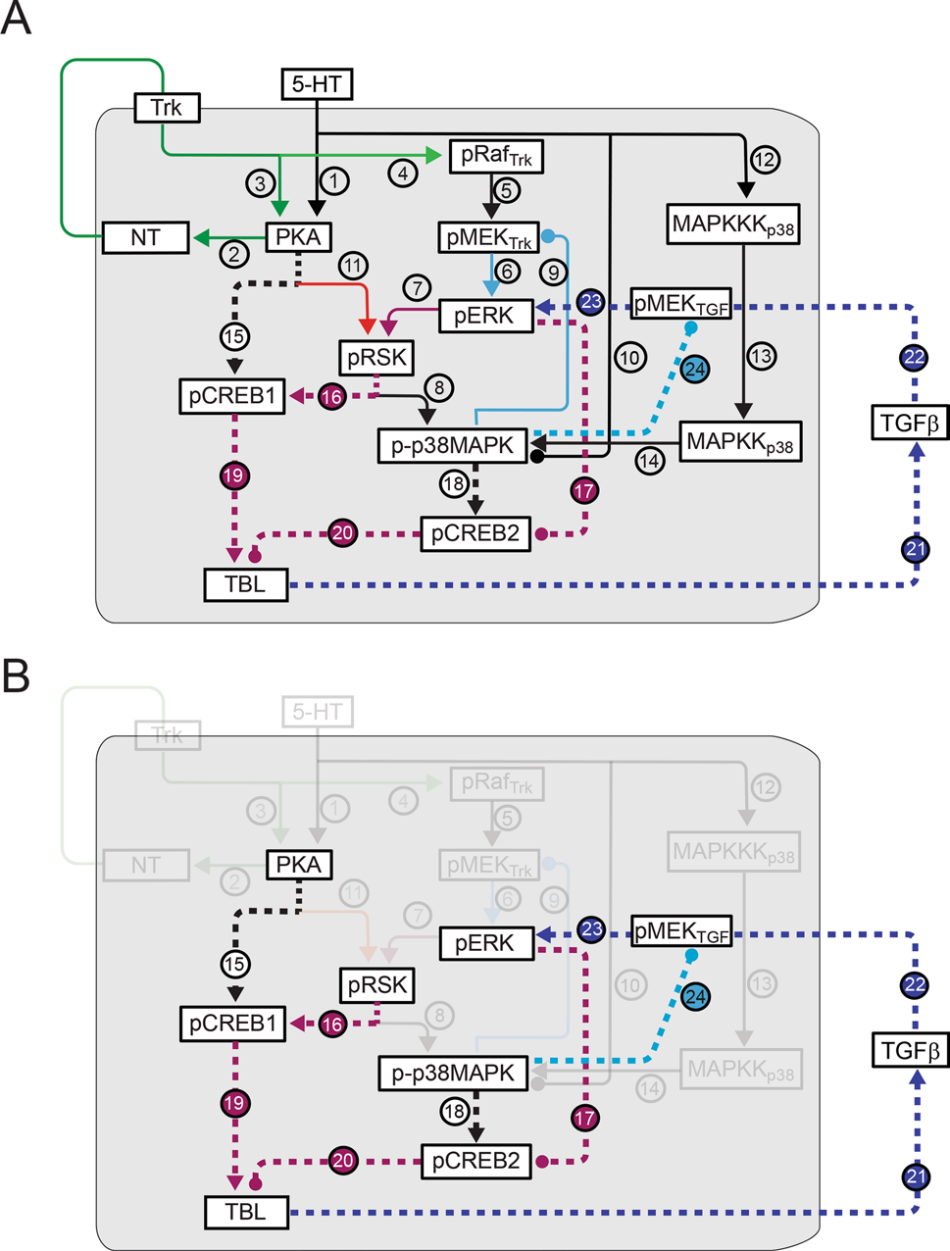
Schematic network including transcription factors CREB1/2, regulated by PKA and MAPK signaling pathways. (**A**) Complete pathways in the extended model. The pathways from Fig. 3A are represented as solid, thinner, lines, and the pathway numbers are indicated with unfilled circles. The newly added pathways are dashed, thicker lines, and pathway numbers are indicated with filled circles. The PKA and MAPK cascades interact to regulate the phosphorylation and activities of CREB1 and CREB2, which regulate the expression of TBL. TBL subsequently activates TGF-β. Violet denotes two ERK – > TBL pathways: ERK- > RSK- > CREB1- > TBL, pathway 7- > 16- > 19); ERK- > CREB2- > TBL, pathway 17- > 20). Arrowheads indicate activation, circular ends indicate repression. (**B**) Network highlighting the addition of new pathways, with the previous pathways made transparent.

**Figure 5:**
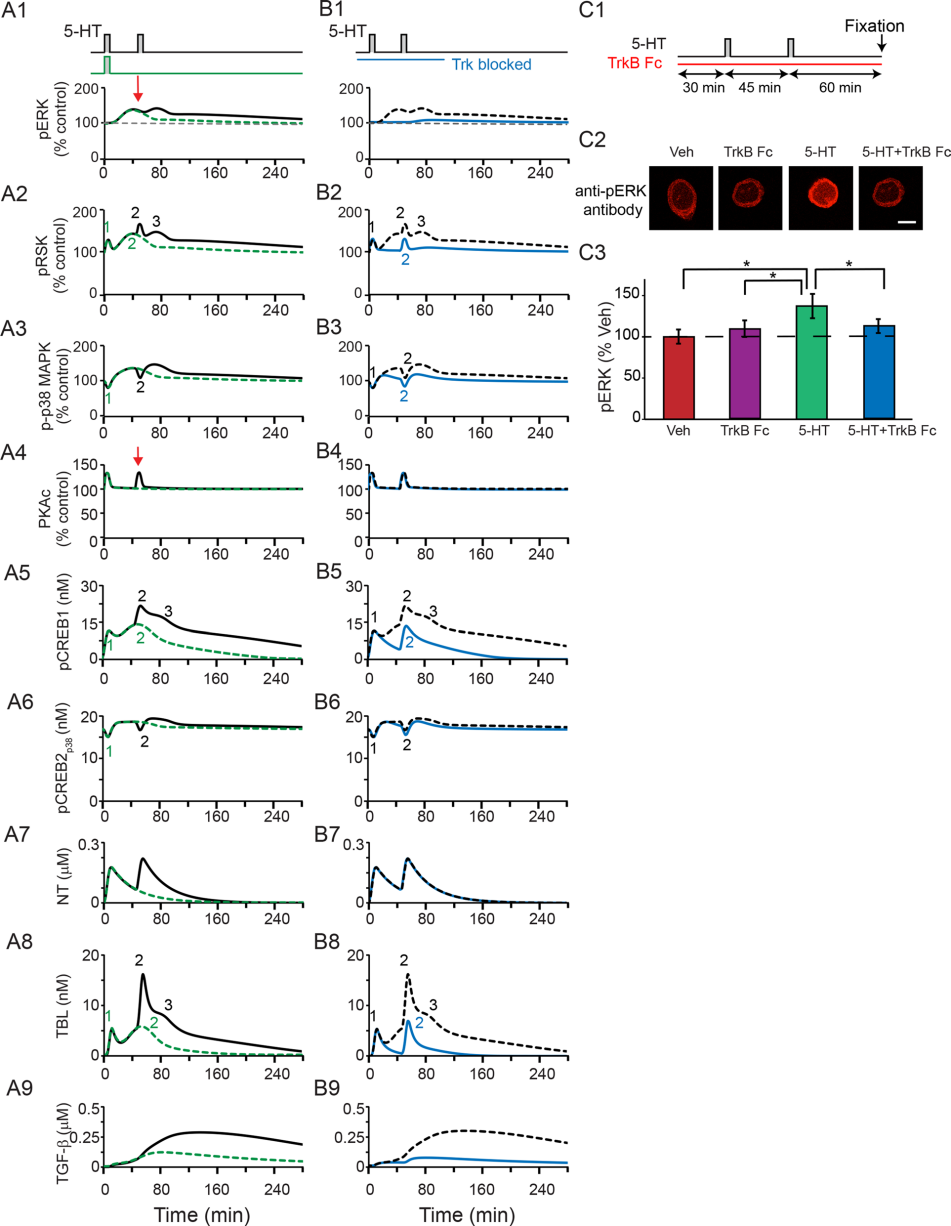
(**A**,**B**) Simulated dynamics of pERK, pRSK, p-p38 MAPK, PKAc, pCREB1, pCREB2_p38_, NT, TBL and TGF-β levels after one or two 5-min pulses of 5-HT with ISI of 45 min, without block of TrkB (**A**), or with block of TrkB applied during both pulses of 5-HT (**B**). (**A**) Black solid curves, simulations after two pulses of 5-HT. Green curves, simulations after one pulse of 5-HT. Red arrows in A1 and A4 represent the overlapped increases of pERK and PKAc after two pulses of 5-HT. (**B**) Blue curves, simulations after two pulses with inhibitors; black dashed curves, control simulations without inhibitors, same as black solid curves in A. Numbers “1”, “2”, and “3” in A2, A5, A8, B2, B5, and B8 represent the waves or late shoulders of increase. Numbers “1”, and “2” in A3, A6, B3, and B6 represent the waves of decrease. (**C**) Empirical validation that TrkB Fc applied during both pulses of 5-HT suppressed the increase of pERK at 1 h post-onset of second pulse of 5-HT (n = 7). Data are represented as mean ± SEM. All scale bars are 40 μm. * p < 0.05.

Five pulses of 5-HT with ISIs of 20 min is a common protocol that induces LTF^20,21,40,41^. However, recent studies suggest two trials of tail shock or tail nerve shocks, each of which releases 5-HT, can also induce LTF and LTM if the ISI is 45 min^25,32^. One trial only induces a transient (< 60 min) increase of MAPK activity whereas two trials yield a sustained increase^17^. Simulations of two pulses of 5-HT using the model of Fig. 3A do not induce a sustained increase of pERK (Fig. S2), suggesting that additional pathways are involved in the sustained increase of MAPK activity during the consolidation phase of LTF. Empirically, a critical difference between a single pulse and two, or more, pulse(s) of 5-HT is the engagement of the TGF-β pathway by multiple pulses^17^. We therefore extended the model to incorporate the TGF-β pathway and its activation of ERK, the transcription factors CREB1/2, and the *Aplysia* tolloid/BMP-1-like protein (TBL) (Fig. 4, Methods). The effects of one pulse of 5-HT in the extended model (Fig. 5A) were essentially identical to results with the simpler model (Fig. 3) in that kinases declined in 1 h (Fig. 5A1-A4, green curves). However, two pulses of 5-HT yielded a sustained increase of pERK, persisting ~ 3 h (Fig. 5A1), as well as sustained increases of pRSK and phosphorylated CREB1 (pCREB1) (Fig. 5A2, A5, black curves).

Two pulses induced two waves of increase in pRSK and pCREB1 (Fig. 5A2 and A5). The peaks of the second waves induced by two pulses were substantially higher than the second waves induced by one pulse (Fig. 5A2 and A5, black vs. green ‘2’). For two pulses, these peaks were followed by late shoulders, which were absent after one pulse (Fig. 5A2 and A5, black ‘3’). With two 5-HT pulses, the enhanced second wave of pRSK was due to the overlap of the delayed increase in pERK after the first pulse with the immediate increase in PKAc after the second pulse (Fig. 5A1 and A4, red arrows), whereas the late shoulder was dependent on the Trk—ERK pathway activated by the second pulse.

Simulations show that two pulses induced two waves of transient decrease in p-p38 MAPK, and in CREB2 activated by p38 MAPK (pCREB2_p38_) (Fig. 5A3 and A6, ‘1’, ‘2’). Two pulses also induced concurrent increases in pRSK and pCREB1 (Fig. 5A2 and A5, ‘1’, ‘2’). The concurrently increased pCREB1 and decreased pCREB2_p38_ substantially elevated TBL and TGF-β (Fig. 5A8, A9). This increase was sufficient to activate the ERK—TGF-β feedback loop (Fig. 4), leading to a sustained increase of pERK (Fig. 5A1).

#### Simulated interaction of the NT/Trk and TGF-β pathways

Kopec et al.^17^ suggested the NT/Trk and TGF-β pathways act independently to regulate discrete phases of MAPK activation, with the NT/Trk pathway responsible for the early activation of ERK and the TGF-β pathway responsible for the late persistent activation of ERK. The simulation results in Fig. 5A replicated the empirical finding that the increase of pERK after one pulse of 5-HT is NT/Trk pathway dependent. After one pulse, TGF-β is increased only slightly (Fig. 5A9, green trace), and there is minimal increase of pERK after 1 h (Fig. 5A1, green trace). Two pulses with ISI of 45 min transiently increased NT (Fig. 5A7, black trace), but increased TGF-β (Fig. 5A9, black trace), and thus pERK (Fig. 5A1, black trace) for ~ 3 h. These simulations suggest that the activation of ERK-TGF-β feedback is necessary for the sustained increase of pERK (Fig. 5A1). The NT/Trk-ERK pathway does not appear sufficient (Fig. S2).

In order to test the necessity of the NT/Trk-ERK pathway, NT was set to zero during both pulses of 5-HT (from 10 min prior to the first pulse to 1 h post-onset the second pulse). In the complete absence of Trk activation, pERK failed to substantially increase 1 h after the second pulse (Fig. 5B1, blue curve). Only two waves of transient increase were induced in pRSK, pCREB1, and TBL (Fig. 5B2, 5, 8), which appear insufficient to activate TGF-β and induce a sustained increase of pERK (Fig. 5B9, blue curve). Thus, based on these simulations, we predict that activation of the NT/Trk pathway plays a role in activating the ERK—TGF-β feedback loop. Only a transient activation, by a single 5-HT pulse, seems to be required because blocking Trk activation during either the first or second pulse of 5-HT failed to block the persistent increase in pERK (Fig. S3). Simulated pERK 1 h after 5-HT treatment was 125% of control (basal level) in the absence of Trk inhibitor; 124% of control if Trk inhibitor was added during the first pulse, and 119% of control if Trk inhibitor was added during the second pulse. These simulation results are qualitatively consistent with empirical findings^17^ indicating that Trk activation during either the first or second pulse of 5-HT failed to block the persistent increase in pERK.

We also simulated the effects of blocking TGF-β activation during the first or second pulse of 5-HT (Fig. S4). Blocking TGF-β activation during the first pulse did not affect the sustained increase in pERK (Fig. S4A, blue curves), whereas blocking TGF-β activation during the second pulse blocked the increase of TGF-β and sustained increase of pERK (Fig. S4B, blue curves). We also compared the effects of Trk and TGF-β inhibitors on the sustained increase of pERK if the inhibitors were added 1 h after 5-HT treatment. TGF-β inhibitor added 1 h after 5-HT decreased TGF-β and reduced pERK to the basal level, whereas Trk inhibitor added 1 h after 5-HT did not affect TGF-β and pERK (Figs. S4C, D, blue curves). These simulation results suggest that the TGF-β feedback loop is a major contributor to the sustained increase of pERK, consistent with the findings of Chin et al.^15^ and Kopec et al.^17^.

#### Empirical test of model prediction

To test the prediction that blocking Trk can block sustained ERK activation, we examined pERK 1 h post-onset of the second pulse in the absence or presence of a human recombinant TrkB antagonist (TrkB Fc) applied throughout two pulses of 5-HT (Fig. 5C1, Methods). Example responses of pERK and summary data are illustrated in Fig. 5C2-5C3. 5-HT alone led to a 40.7 ± 13.9% increase in pERK. In the presence of TrkB Fc, 5-HT led to only a 10.5 ± 7.7% increase in pERK, thus sustained ERK activation was inhibited. Pairwise comparisons (SNK) following a RM ANOVA revealed that the 5-HT alone group was significantly different from the other three groups (Table S1). These data support the prediction that some activation of the NT/Trk–ERK pathway is needed to activate the ERK–TGF-β feedback loop, even though the subsequent sustained increase of pERK is dependent on TGF-β.

#### The roles of the PKA-RSK and RSK-p38 MAPK pathways

**Figure 6:**
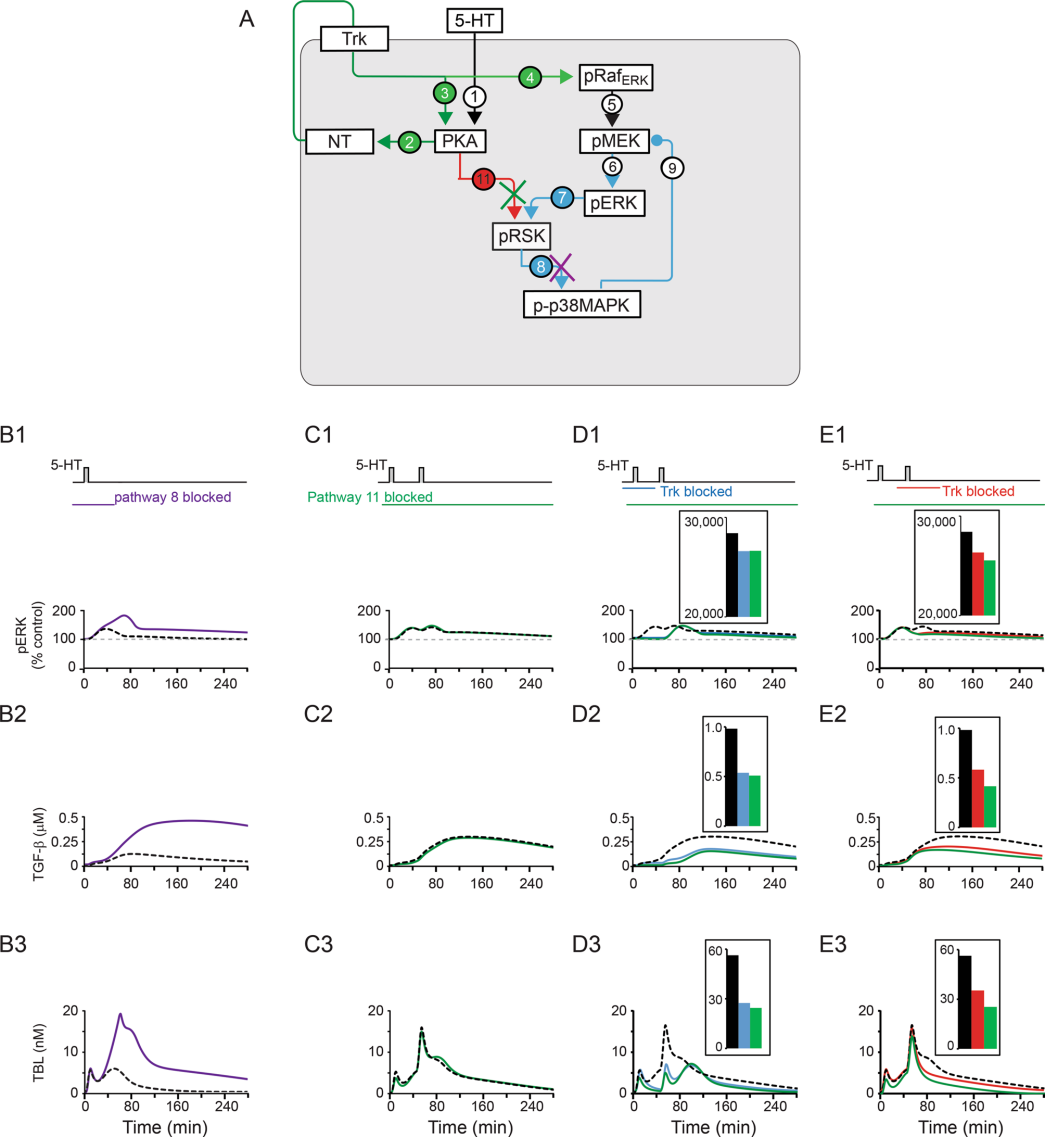
Simulated dynamics of pERK, TGF-β, and TBL after one pulse of 5-HT, with or without suppression of pathway 8 (purple ‘x’) or pathway 11 (green ‘x’) in (**A**). (**B**) Dynamics of pERK, TGF-β, and TBL after one pulse of 5-HT with blocking of pathway 8 (purple curves). Black dashed curves are control simulations with no suppression. (**C–E**) Dynamics of pERK, TGF-β, and TBL after two pulses of 5-HT (ISI = 45 min) with block of pathway 11 in the absence of block of the Trk pathway (**C**, green curve), or in the presence of Trk inhibitor during either the first (**D**, green curve) or second pulse (**E**, green curve) of 5-HT. Also shown are the effects of only blocking the Trk pathway during the first (**D**, blue curve) or second (**E**, red curve) pulse of 5-HT. Black dashed curves are control simulations. The histograms in boxes in **D** and **E** denote the integral values of pERK, TGF-β, and TBL between 50 and 280 min, with bar colors matching the corresponding curves.

Zhang et al.^11^ suggest that the increase in pERK after one pulse could be substantially prolonged by inhibition of p38 MAPK. Thus, we blocked pathway 8 to simulate the effects of p38 MAPK inhibition (Fig. 6A). In this case one pulse of 5-HT sufficed to induce a sustained increase of pERK, TBL and TGF-β (Fig. 6B). This simulation suggests p38 MAPK activation might act as a constraint to prevent over-activation of the ERK cascade and subthreshold induction of LTF.

We also investigated the contribution of the PKA-RSK pathway to the dynamics of downstream processes. Blocking activation of RSK by PKA (blocking pathway 11 in Fig. 6A throughout both pulses of 5-HT) had little effect on the activation of pERK in the absence of Trk inhibitor (Fig. 6C, green vs. black curves), or when Trk was blocked during the first pulse (Fig. 6D, blue vs. green curves). However, a greater role of the PKA-RSK pathway 11 was revealed when Trk was blocked during the second pulse (Fig. 6E), suggesting a compensatory role of pathway 11 in maintaining the sustained increase of pERK. Blocking Trk alone reduced TBL which in turn reduced TGF-β and pERK, but TBL, TGF-β and pERK were reduced further when activation of RSK by PKA (pathway 11) was also blocked (Fig. 6E1-3, green vs. red vs. black curves, the integral histograms of pERK and TGF and TBL between 50 and 280 min). Blocking Trk during the first pulse revealed a similar contribution of pathway 11 to TGF and TBL (Fig. 6D2-3, green vs. blue curves, the integral histograms of TGF and TBL between 50 and 280 min), but weaker than after blocking Trk during the second pulse, not sufficient to substantially reduce pERK (Fig. 6D1, green vs. blue curves, the integral histograms of pERK between 50 and 280 min). The dynamics of pCREB2_p38_ help explain these differences. After the first pulse, 5-HT decreased pCREB2_p38_ (Fig. 5A6, black curve, ‘1’, pathway 10 → 18). Thus, when the Trk pathway was blocked at the first pulse, even in the absence of pathway 11, pCREB2_p38_ decreased after this pulse, and therefore sufficient TBL was induced after the second pulse to activate TGF-β. Thus, the sustained increase of pERK was not substantially reduced (Fig. 6D). In contrast, after the second pulse, pCREB2_p38_ does not decrease as much (Fig. 5A6, black curve, ‘2’), so that the induction of sufficient TBL is more dependent on pathway 11 and its activation of pCREB1 through RSK.

#### Simulations of two-pulse and five-pulse protocols with different ISIs

We previously used a simplified model of PKA and MAPK pathway dynamics during the induction of LTF to determine, through permutations of ISIs, a protocol that maximized simulated overlap between PKA and ERK activition^28^. The overlap between PKA and ERK activities was quantified by their multiplicative activation of a phenomenological variable *inducer*^28^. This “Enhanced” protocol of irregularly spaced 5-HT pulses was subsequently verified empirically to produce greater levels of LTF and LTM than did the “Standard” protocol of regularly spaced pulses^28^. To examine whether the current model has similar predictive ability, we compared the peak levels of *inducer* activated by the Enhanced and Standard protocols.

$$inducer \, = { (}[PKA_{C} ] - [PKA_{C} ]_{basal} )([ERK^{pp} ] - [ERK^{pp} ]_{basal} )$$

**Figure 7:**
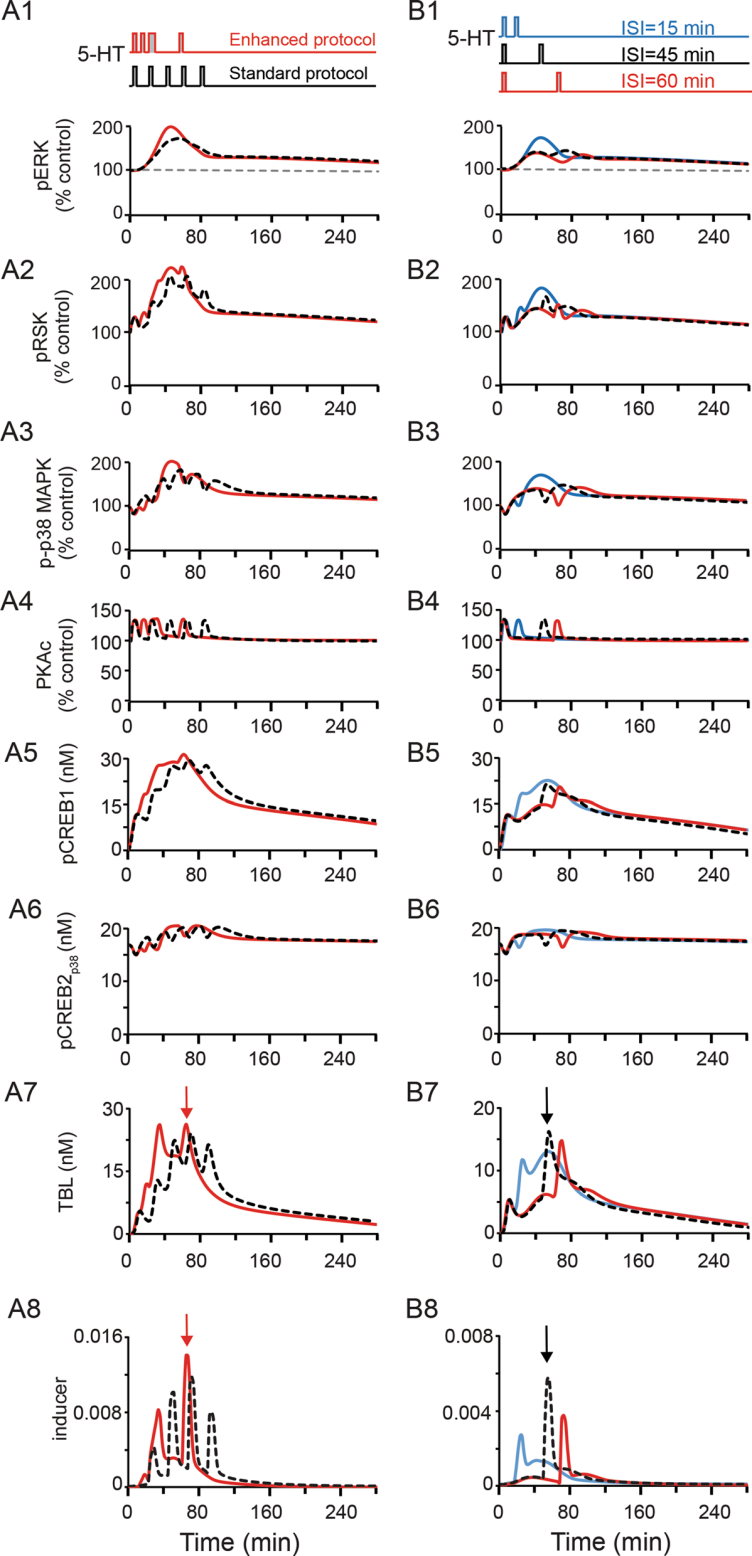
Simulated dynamics of kinases, pCREB1, pCREB2_p38_, TBL and *inducer* levels after five pulses of 5-HT using the Standard protocol (**A**, black curves), or Enhanced protocol (**A**, red curves); or two pulses of 5-HT with ISI of 15 min (**B**, blue curves), 45 min (**B**, black curves) or 60 min (**B**, red curves). Arrows represents peak *inducer* levels.

With the current model, the Enhanced protocol still produced a better overlap of kinases and a higher peak level of *inducer* than did the Standard protocol (Fig. 7A8, red arrow). Moreover, the Enhanced protocol also produced a higher peak level of TBL than did the Standard protocol (Fig. 7A7, red arrow). Thus, the molecular network of PKA and MAPK cascades in the revised model produced consistent dynamics with five pulses of 5-HT, as compared to the previous model^28^.

In addition, LTM can be induced empirically by two learning trials separated by an ISI of 45 min, but not by ISIs of 15 or 60 min^25,32^. We therefore simulated the overlap of PKA and ERK activities generated by two pulses of 5-HT with ISIs of 15, 45, and 60 min (Fig. 7B). Two simulated pulses of 5-HT with an ISI of 45 min produced a higher peak level of ‘*inducer*’ than did ISIs of 15 or 60 min (Fig. 7B8, black arrow). Moreover, two simulated pulses of 5-HT with an ISI of 45 min produced a higher peak level of TBL than did ISIs of 15 or 60 min (Fig. 7B7, black arrow). Thus, the molecular network of PKA and MAPK cascades in the revised model suggests an explanation for why two trials separated by ISI of 45 min can induce LTM.

## Discussion

### The role of the PKA-RSK pathway

The PKA-mediated regulation of RSK, independent of ERK, has not been reported in previous studies. We used two PKA inhibitors to investigate the possibility that phosphorylation of RSK can be regulated by PKA (Fig. 2). Simulations of one and two pulses of 5-HT suggest the PKA-RSK pathway may not be necessary for sustained increase of pERK under normal conditions (Fig. 6). However, these simulations illustrated that when the NT-Trk-ERK pathway was blocked, the PKA-RSK pathway did contribute to the sustained increase of pERK and TGF-β after two pulses, indicating that the PKA-RSK pathway may provide an alternate pathway for induction of LTF if the NT-Trk-ERK pathway is compromised. Should these interactions be observed in mammalian neurons, the PKA-RSK pathway may serve as a possible therapeutic target for deficits of memory due to impairment of neurotrophin expression.

### The role of the RSK-p38 MAPK pathway

Zhang et al.^11^ found a previously unreported activation of p38-MAPK by 5-HT. We found that this increase of p-p38 MAPK at 45 min can be blocked by RSK inhibition (Fig. 3D), suggesting an ERK → RSK → p38 MAPK pathway, and possibly explaining why the MEK1/2 inhibitor U0126 suppresses the increase of p-p38 MAPK^11^ (Fig. 3A, pathway 6 → 7 → 8).

Increased p-p38 MAPK due to the activation of the ERK → RSK → p38 MAPK pathway may negatively feed back to the MEK/ERK pathway (Fig. 3A, pathway 9). Simulations suggest that if the RSK-p38 MAPK pathway is blocked, one pulse of 5-HT is sufficient to yield prolonged activation of pERK (Fig. 6A). Thus, the RSK-p38 MAPK pathway may serve as a key constraint to block the induction of LTF after a single, subthreshold training session. Repeated exposure would overcome the negative feedback loop, transforming short-term facilitation to LTF.

The RSK-p38 MAPK pathway found in this study, if present in mammals, may be in part responsible for deficits in synaptic plasticity observed in RASopathies^43,44^. Aberrant constitutive activation of the Ras → ERK signaling pathway impairs long-term potentiation (LTP) and learning^45^. The model predicts that constitutive ERK activation leads to RSK and p38 MAPK activation (Fig. 3A, pathway 7 → 8). Constitutive p38 MAPK activation could impair learning by suppressing BDNF-dependent late LTP^46^. Thus, p38 MAPK inhibitors might be considered as possible treatments for RASopathies. Another genetic mutation, Coffin-Lowry Syndrome, is caused by X-linked mutations in *rsk2*. In mice, this deficiency impairs fear memory consolidation and CREB phosphorylation^47–49^. However, to our knowledge, the role of RSK in mammalian long-term synaptic plasticity remains inconclusive, and its elucidation could be complicated by the potential ERK—RSK–p38 MAPK–ERK negative feedback loop. A reduction in RSK would reduce CREB phosphorylation, which would tend to impair LTP. However, a reduction in RSK would also reduce the activation of p38 MAPK (Fig. 3A, pathway 8), which suppresses negative feedback to MEK (Fig. 3A, pathway 9), enhances pERK, and possibly augments LTP. In the hippocampus of *mrsk2*_KO mice, phosphorylation of ERK1/2 is abnormally increased, suggesting the negative feedback pathway from RSK to ERK does exist in mammalian neurons^50^.

### The role of the ERK-TBL- TGF-β positive feedback loop

To induce a sustained increase of pERK, the model includes the ERK—TBL—TGF-β positive feedback loop^11,14,15,17,42^. Empirical results supported the model prediction that a transient increase in activity of a NT-Trk-ERK-RSK pathway is necessary to activate the ERK—TBL—TGF-β feedback loop (Fig. 5). The apparent key role of this feedback loop suggests that interventions to enhance this feedback may enhance LTF and LTM.

### Model insights, limitations, and future directions

Signaling cascades underlying memory and most other biological phenomena are complex, and computational modeling is necessary to reach a deeper understanding than can otherwise be achieved. The model developed here represents the most detailed attempt to delineate the complex interactions between PKA and MAPK pathways responsible for the induction of LTF, LTEE, and LTM. It fits the available data, consolidates empirical findings, makes predictions, and can serve as a dynamic database of SN signaling cascades. Simulations of the model provide insights into the complex dynamics underlying the induction of LTEE and LTF, which is difficult for an empirical approach alone to delineate. Our simulations suggest:

- The delayed activation of ERK after a single training stimulus is due to the slow dynamics of growth factors.
- The apparent independence of two growth factor pathways, Trk and TGF-β, is due to Trk-independent pathways that increase active CREB1 and decrease active CREB2, activating TGF-β expression.
- Feedback and feedforward loops play critical roles in the dynamics of kinases. The positive ERK- TGF-β feedback loop leads to sustained increase of pERK.
- The positive PKA—RSK feedforward loop helps enhance RSK activity and induces the ERK-TGF-β feedback loop when the Trk-ERK-RSK pathway is blocked.
- The negative ERK–p38 MAPK feedback loop suppresses the increase of pERK 1 h after a single training stimulus, thus adding a constraint to the efficiency of training protocols.

Some aspects of SN function remain to be included in the model. For example, the model lacks separate synaptic and somatic compartments^17^, CREB or ELAV–regulated gene expression^22,23,51,52^, and synaptic structural changes accompanying LTF^53^. The model also does not include processes in the postsynaptic motor neuron that contribute to LTF^18,54^.

Despite these limitations, the model can describe many features of SN plasticity. Because many signaling pathways and transcription factors involved in late LTP and memory are conserved between *Aplysia* and mammals^19^, this model is expected to provide insights into mechanisms underlying late LTP and aid in the development of strategies for enhancing memory with pharmacological manipulations and training protocols that resonate with the dynamics of the underlying biochemical cascades.

## Materials and methods

### Empirical methods

#### Neuronal cultures

All experiments used primary cultures of identified sensory neurons (SNs) from *Aplysia californica* (following the guidelines of NIH *Aplysia* resource facility, University of Miami, Miami, FL). *Aplysia* are hermaphrodites. Animals were maintained in circulating artificial seawater at 15 °C. SNs were isolated from the ventral-caudal cluster of the pleural ganglion from 60–100 gm *Aplysia* according to conventional procedures^11,23,28,55^. Each dish of SN cultures was plated with 5–10 SNs. SNs were allowed to grow for 5–6 days at 18 °C before experiments begun, and the growth medium was replaced at least 2 h prior to treatments with a solution of 50% L15 and 50% artificial seawater (ASW; 450 mM NaCl, 10 mM KCl, 11 mM CaCl2, 29 mM MgCl2, 10 mM HEPES at pH 7.6).

#### Immunofluorescence analysis

Immunofluorescence procedures for SNs followed those of Zhang et al.^11^. Briefly, at different time points after 5-HT treatment, cells were fixed in a solution of 4% paraformaldehyde in PBS containing 20% sucrose. After three 5-min rinses in PBS, fixed cells were blocked for 30 min at room temperature in a solution of Superblock buffer (Pierce), 0.2% Triton X-100, and 3% normal goat serum, and subsequently incubated overnight at 4 °C with anti-cAMP protein kinase catalytic subunit (anti-PKA catalytic subunits (anti-PKAc), Abcam, Cat # ab76238, RRID: AB_1523259, 1:2,000 dilution), anti-phosphorylated ERK (anti-pERK, Cell Signaling, Cat # 4370, RRID: AB_2315112, 1:400), anti-phosphorylated RSK (anti-pRSK, Cell Signaling, Cat # 9346, RRID: AB_330795, 1:400), or anti-phosphorylated p38 MAPK rabbit antibody (anti-p-p38 MAPK, Cell Signaling, Cat # 4511, RRID: AB_2139682, 1:400). After primary antibody incubation, secondary antibody (goat anti-rabbit secondary antibody conjugated to Rhodamine Red, Jackson ImmunoResearch Lab, Catalog#: 111–295-144, RRID: AB_2338028, 1:200) was applied for 1 h at room temperature. Cells were then mounted using Mowiol 4–88 (SigmaAldrich). The intensity of staining in SNs was quantified in images obtained with a Zeiss LSM800 confocal microscope using a 63 × oil-immersion lens. A z-series of optical sections through the cell body (0.5 μm increments) was taken, and the section through the middle of the nucleus was used for analysis of mean fluorescence intensity of the whole cell with ImageJ-win64 software (NIH). All the neurons on each coverslip were analyzed, and measurements from these neurons were averaged. All experiments were performed in a blind manner so that the investigator analyzing the images was unaware of the treatment the SNs received. The number of samples (n) reported in Results indicates numbers of dishes assessed.

#### Experimental design

For one pulse of 5-HT treatment, 5 min 50 µM 5-HT (Sigma) was applied to SNs. Dishes of SNs cultured from the same animals were paired for all the 5-HT treatments. One dish received a solution consisting of 50% isotonic L15 and 50% artificial seawater (L15-ASW) as vehicle control (Veh). The other received the same solution with the addition of 5-HT. The experimenter was blind to the identity of the treatments.

In the experiments to measure the time course of phosphorylated RSK (pRSK) after one pulse of 5-HT, one of each paired dish was fixed for immunofluorescence immediately after 5-HT, or incubated in L15/ASW after wash off of 5-HT until fixation at 15, 45 and 60 min after treatment onset. The remaining dish served as a time-matched Veh control. For each pair of dishes measured at the same time point, the averaged level of pRSK from the dish receiving 5-HT was compared to the averaged pRSK from the Veh control.

In the experiments to measure the time course of PKA catalytic subunits (PKAc) after one pulse of 5-HT, one of each paired dish was fixed for immunofluorescence immediately after 5-HT, or incubated in L15/ASW after wash off of 5-HT until fixation at 15 and 45 min after onset of 5-HT. The remaining dish served as a time-matched Veh control. For each pair of dishes measured at the same time point, the averaged level of PKAc from the dish receiving 5-HT was compared to the averaged PKAc from the Veh control.

Application of all the inhibitors began 30 min prior to 5-HT treatment and continued during treatment. After 5-HT was washed out, inhibitors remained present until fixation for immunofluorescence, to ensure that inhibitors were given sufficient time to penetrate the cells and affect the activities of kinases.

To examine the specificity of anti-PKAc antibody, 10 μM cAMP inhibitor Rp-cAMP (Calbiochem) was applied to SN cultures 30 min before and then concurrently with 20 min 5-HT treatment. Four dishes of SNs from the same animals were used for each experiment. Each dish was given a different treatment, either: **1)** 50 μM 5-HT alone; **2)** 10 μM Rp-cAMP alone; **3)** 5-HT + Rp-cAMP; or **4)** Veh alone.

To examine the effects of PKA activity on pERK and pRSK, 10 μM KT5720 (Sigma) or 10 μM cAMP inhibitor Rp-cAMP (Calbiochem) was applied to SN cultures 30 min before and then concurrently with 5-HT treatment. At this concentration, KT5720 inhibits PKA activity in *Aplysia* without affecting basal synaptic strength^40^. In preliminary experiments (Fig. 1B), 10 μM cAMP inhibitor Rp-cAMP inhibited the increase of PKA activity 5-HT without affecting basal activity. Four dishes of SNs from the same animals were used for each experiment. Each dish was given a different treatment, either: **1)** 50 μM 5-HT alone; **2)** 10 μM KT5720 or Rp-cAMP alone; **3)** 5-HT + KT5720 or 5-HT + Rp-cAMP; or **4)** Veh alone.

To examine the effects of MEK/ERK activity on pRSK immediately after 5-HT, 20 μM U0126 (Cell Signaling) was applied to SN cultures 70 min before and then concurrently with 5-HT treatment. With this concentration and duration, in preliminary experiments, U0126 inhibited the increase of ERK activity 45 min post-onset of 5-HT without affecting basal activity. Four dishes of SNs from the same animals were used for each experiment. Each dish was given a different treatment, either: **1)** 50 μM 5-HT alone; **2)** 10 μM U0126 alone; **3)** 5-HT + U0126; or **4)** Veh alone.

To examine the effects of pRSK on p-p38 MAPK, 1–2 μM BI-D1870 (Santa Cruz), was applied to SN cultures 30 min before and then concurrently with 5-HT treatment. Low concentrations of BI-D1870 were used because a higher concentration would affect basal phosphorylation of CREB1 in the absence of 5-HT treatment^23^. Four dishes of SNs from the same animals were used for each experiment. Each dish was given a different treatment, either: **1)** 50 μM 5-HT alone; **2)** BI-D1870 alone; **3)** 5-HT + BI-D1870; or **4)** Veh alone.

To examine the effects of Trk on pERK,10 μg/ml human recombinant TrkB antagonist, TrkB-Fc chimera (TrkB Fc) (R&D Systems), was applied to SN cultures 30 min before, during, and after 5-HT until SNs were fixed. In preliminary experiments, at this concentration, TrkB Fc inhibited the increase of pERK at 45 min post-onset of one pulse of 5-HT in isolated *Aplysia* SNs without affecting basal activity. Four dishes of SNs from the same animals were used for each experiment. Each dish was given a different treatment, either: 1) 5-HT alone; 2) TrkB Fc alone; 3) 5-HT + TrkB Fc; or 4) Veh alone.

#### Statistical analyses

At least five animals were used in each experiment. SigmaPlot version 11 (Systat Software) was used for statistical analyses. Before applying other statistical tests, Shapiro–Wilk Normality and Equal Variance tests were performed. In the experiments to compare pRSK or PKAc between paired Veh and 5-HT treatment groups, a paired t-test with Bonferroni corrections was used for comparison between two groups if data passed normality and equal variance tests at all time points. Otherwise, a Wilcoxon Signed Rank Test (WSRT) with Bonferroni corrections was used. Thus, the WSRT with Bonferroni corrections was used for comparison of pRSK immunoreactivity between paired Veh and 5-HT treatment groups at all time points because data for the normality variance test failed at one time point (Fig. 1A). For measuring multiple time points of PKAc after 5-HT (Fig. 1B), the paired t-test with Bonferroni corrections was used for comparison between paired Veh and 5-HT treatment groups at all time points. Adjusted p values after Bonferroni corrections were used to represent statistical significance.

In the experiments to make multiple comparisons between groups treated with 5-HT and inhibitors, repeated measures one-way (RM) ANOVA and the post hoc Student-Newman–Keuls (SNK) method were used on raw data (Fig. 2A–D, and 5C). For data displaying a non-normal distribution (Fig. 2E), repeated measures analysis of variance on ranks and the post hoc SNK method were used. Repeated measures ANOVA was used here because we distributed the SNs from a single animal across the different groups, so that samples in each group were not independent.

Data from all experiments were presented as means ± SEM, and p < 0.05 was considered to represent statistical significance. All data are available upon request.

### Computational model of kinase signaling pathways induced by 5-HT

A particular strength of combined empirical and computational approaches has been their iterative combination, allowing for continual refinement of models, and concurrent empirical testing of hypotheses developed via simulations. The computational model in this study is revised from previous products of this approach^11,28,55^. The parameters of these models have been constrained by available empirical data and predictions of the models have been validated by empirical studies. The parameters were further constrained in this study and new predictions made in this study were subsequently empirically validated.

#### Modeling the PKA and MAPK signaling cascades

The equations describing the PKA and MAPK cascades are adapted from the models of Pettigrew et al.^56^, Zhang et al.^28^ and Zhang et al.^11^. Equations characterizing the new elements that were not in the previous models, and revisions to previous models (grey dashed lines in Fig. 3A), were based on empirical data in recent studies^11,17,18,23^ and the present study.

#### ERK pathway

The activation of ERK was modeled as a cascade with sequential activation of the kinases Raf, MEK, and ERK (Fig. 3A, pathway 5 → 6). The ordinary differential equations describing the activation of Raf, MEK, and ERK (Eqs. 1–8) are similar to those in Zhang et al.^11^ and Pettigrew et al.^56^, but modifications were made to make the model more biologically realistic. The discrete delay for ERK activation used in Zhang et al.^28^ was replaced by equations that describe 5-HT activation of Raf via the PKA/*Aplysia* neurotrophin (NT)/presynaptic receptor Trk-like pathway (Fig. 3A, pathway 1 → 2 → 4) (Eq. 1, Kopec et al.^17^; Jin et al.^18^), resulting in slow activation of the Raf-MEK-ERK pathway. The detailed dynamics of the NT/Trk pathway activated by 5-HT are unclear. Therefore, to simplify the model, a single new variable *NT* (Eq. 15) is used to represent the activity of the NT/Trk pathway.

*[ERK*^*pp*^*]* corresponds to the pERK level measured by immunofluorescence (*i.e.,* ERK activity). The equations describing the ERK pathway are,

1 $$\frac{{d[Raf_{TrkB}^{p} ]}}{dt} = (k_{basal,Raf} + k_{f,Raf} [NT])[Raf_{TrkB} ] - k_{b,Raf} [Raf_{TrkB}^{p} ]$$

2 $$[Raf_{TrkB} ] = [Raf_{TrkB} ]_{total} - [Raf_{TrkB}^{p} ]$$

3 $$\frac{{d[MEK_{TrkB} ]}}{dt} = \frac{{k_{{_{{b,MEK_{TRKB} }} }} [MEK_{TrkB}^{p} ]}}{{[MEK_{TrkB}^{p} ] + K_{MEK,2} }} - \frac{{k_{f,MEK} [Raf_{TrkB}^{p} ][MEK_{TrkB} ]}}{{[MEK_{TrkB} ] + K_{MEK,1} }}$$

4 $$\frac{{d[MEK_{TrkB}^{pp} ]}}{dt} = \frac{{k_{f,MEK} [Raf_{TrkB}^{p} ][MEK_{TrkB}^{p} ]}}{{[MEK_{TrkB}^{p} ] + K_{MEK,1} }} - \frac{{k_{{b,MEK_{TRKB} }} [MEK_{TrkB}^{pp} ]}}{{[MEK_{TrkB}^{pp} ] + K_{MEK,2} }}$$

5 $$[MEK_{TrkB}^{p} ] = [MEK_{TrkB} ]_{total} - [MEK_{TrkB} ] - [MEK_{TrkB}^{pp} ]$$

6 $$\frac{d[ERK]}{{dt}} = \frac{{k_{b,ERK} [ERK^{p} ]}}{{[ERK^{p} ] + K_{ERK,2} }} - \frac{{k_{f,ERK} [MEK_{TrkB}^{pp} ][ERK]}}{{[ERK] + K_{ERK,1} }}$$

7 $$\frac{{d[ERK^{pp} ]}}{dt} = \frac{{k_{f,ERK} [MEK_{TrkB}^{pp} ][ERK^{p} ]}}{{[ERK^{p} ] + K_{ERK,1} }} - \frac{{k_{b,ERK} [ERK^{pp} ]}}{{[ERK^{pp} ] + K_{ERK,2} }} \,$$

8 $$[ERK^{p} ] = [ERK]_{total} - [ERK] - [ERK^{pp} ]$$

Applying the p38 MAPK inhibitor SB203580 (SB) for 1 h after one pulse of 5-HT prevents the normal return of pERK to the basal level at 60 min, possibly via releasing the inhibition of MEK1 by p38 MAPK^57^. Therefore in the model, MEK, ERK, RSK, and p38 MAPK form a negative feedback loop (Fig. 3A, pathway 6 → 7 → 8 → 9 → 6). Equations 9–10 below, used to simulate the inhibitory effect of p38 MAPK on MEK, are modified from Zhang et al.^11^ (Fig. 3A, pathway 9). The variable *E*_*p38-MEK*_ in Eq. (10) represents the protein phosphatases activated by p-p38 MAPK, as described in Zhang et al.^11^ and Westermarck et al.^57^. In Eq. 9, these activated protein phosphatases increase the rate for MEK deactivation, $k_{{b,MEK_{TRKB} }}$(Eqs. 3, 4 above). SB increases pERK after 5-HT treatment, but does not affect the basal level of pERK (Fig. 3 in Zhang et al.^11^). Also, the transient decrease of p-p38 MAPK immediately after 5-HT does not lead to an increase of pERK^11,25^. Therefore, we hypothesized that p38 MAPK only inhibits the MEK/ERK pathway when *MEK*^*PP*^ and *p38 MAPK*^*PP*^ are higher than the basal levels.

Step functions, denoted as *()*^+^, are used in this study to represent ‘lack of basal effects.’ For example, Eq. (10) uses $([P38^{pp} ] - [P38^{pp} ]_{basal} )([P38^{pp} ] - [P38^{pp} ]_{basal} )^{ + }$. $([P38^{pp} ] - [P38^{pp} ]_{basal} )([P38^{pp} ] - [P38^{pp} ]_{basal} )^{ + }$ = 0 when *[P38*^*pp*^*]* is equal to or lower than *[P38*^*pp*^*]*_*basal,*_ so that the effect of p38 MAPK on MEK is 0, and $([P38^{pp} ] - [P38^{pp} ]_{basal} )([P38^{pp} ] - [P38^{pp} ]_{basal} )^{ + }$ = *[P38*^*pp*^*]*—*[P38*^*pp*^*]*_*basal*_ when *[P38*^*pp*^*]* is higher than *[P38*^*pp*^*]*_*basal*_ so that p38 MAPK inhibits MEK. These expressions implement the assumption that if p38 MAPK is at or below a basal level, p38 MAPK activity is not strong enough to substantially affect MEK activity. Likewise, Eq. (9) uses $([MEK_{TrkB}^{pp} ] - [MEK_{TrkB}^{pp} ]_{basal} )^{ + }$, a step function, which is zero when $[MEK_{TrkB}^{pp} ]$ is lower than $[MEK_{TrkB}^{pp} ]_{basal}$.

9 $$k_{{b,MEK_{TRKB} }} = k_{b,MEK\_basal} + k_{b,MEK\_p38} ([MEK_{TrkB}^{pp} ] - [MEK_{TrkB}^{pp} ]_{basal} )^{ + } [E_{p38 - MEK}]$$

10 $$\frac{{d[E_{p38 - MEK} ]}}{dt} = k_{EP38,MEK} ([P38^{pp} ] - [P38^{pp} ]_{basal} )([P38^{pp} ] - [P38^{pp} ]_{basal} )^{ + } - k_{d,EP38,MEK} [E_{p38 - MEK}]$$

#### PKA and NT/Trk pathways

The differential equations describing the increase in *cAMP* and consequent *PKA* activation after 5-HT (Eqs. 11–14) are similar to those in Zhang et al.^28^ and Pettigrew et al.^56^, except that the NT/Trk pathway increases *cAMP* (Eq. 11).^18^. Inactive PKA holoenzyme (PKA_RC_) consists of regulatory (PKA_R_) and catalytic (PKA_C_) subunits. New differential equations describe activation of the NT/Trk pathway (Eq. 14, Fig. 3A, pathway 1 → 2) and its interaction with the PKA and ERK pathways, based on the empirical results in this study and in Jin et al.^18^ (Fig. 3A, pathway 1 → 2 → 4 → 5 → 6 to activate ERK and 1 → 2 → 3 → 2 to feed back to activate PKA). For simplicity, the NT/Trk pathway is represented by a single variable NT in Eq. 15. In response to 5-HT treatment (Fig. 3A, pathway 1) or *NT* activation (Fig. 3A, pathway 3, a feedback loop described by Jin et al.^18^), *cAMP* increases and binds to PKA_R_, leading to the release of active PKA_C_. *NT* in Eq. 15 corresponds to the activated NT/TrkB pathway, which will activate Raf in Eq. 1 (Fig. 3A, pathway 4).

11 $$\frac{d[cAMP]}{{dt}} = \lambda (\frac{{[5{ - }HT]}}{{[5{ - }HT] + K_{5HT} }} + \frac{[NT]}{{[NT] + K_{TrkB} }}) - k_{b,cAMP} [cAMP] + cAMP_{bas}$$

12 $$\frac{{d[PKA_{RC} ]}}{dt} = k_{b,PKA} [PKA_{C} ][PKA_{R} ] - k_{f,PKA} [PKA_{RC} ][cAMP]^{2}$$

13 $$\frac{{d[PKA_{R} ]}}{dt} = k_{f,PKA} [PKA_{RC} ][cAMP]^{2} - k_{b,PKA} [PKA_{C} ][PKA_{R} ]$$

14 $$\frac{{d[PKA_{C} ]}}{dt} = k_{f,PKA} [PKA_{RC} ][cAMP]^{2} - k_{b,PKA} [PKA_{C} ][PKA_{R} ]$$

15 $$\begin{gathered} \frac{d[NT]}{{dt}} = k_{f,NT} \frac{{[PKA_{C} ] - [PKA_{C} ]_{basal} }}{{[PKA_{C} ] - [PKA_{C} ]_{basal} + K_{PKAc,NT} }}([PKA_{C} ] - [PKA_{C} ]_{basal} )^{ + } \hfill \\ \, - k_{b,NT} [NT] \hfill \\ \end{gathered}$$

#### p38 MAPK pathway

The differential equations describing the activation of p38 MAPK (Eqs. 16–25) were modified from Zhang et al.^11^. A new RSK-dependent pathway was added to phosphorylate p38 MAPK. Combining the empirical results in this study and in Zhang et al.^11^, we hypothesized that 5-HT engages separate pathways to inhibit *vs.* to activate p38 MAPK. The mechanism underlying the transient decrease of p38 MAPK activity immediately after 5-HT is unclear. In the model, following the assumption we used in Zhang et al.^11^, 5-HT directly inhibits the phosphorylation rate of p38 MAPK, by decreasing the rate constant *k*_*f,p38*_ (Eqs. 24–25) (Fig. 3A, pathway 10). The subsequent activation of p38 MAPK by 5-HT is via two pathways. One is via RSK (Eqs. 22–24; Fig. 3A, pathway 8), based on the empirical findings in this study. The other is a Raf_p38_/MEK_p38_ pathway (Fig. 3A, pathway 12 → 13 → 14)^11^, analogous to the MKK3/6 pathway in mammalian cells^58,59^. Whether or how 5-HT activates the MKK3/6 pathway in *Aplysia* requires further investigation. As in Zhang et al.^11^, we used direct activation of *Raf*_*p38*_ by 5-HT to represent this pathway (Eqs. 16).

As in Zhang et al.^11^, $[E_{5 - HT} ]$ in Eqs. (24, 25) represents the transient suppressing effect of 5-HT on the phosphorylation of p38 MAPK (Fig. 3A, pathway 10). This transient decrease of p-p38 MAPK immediately after 5-HT has been reported in previous studies^11,29^. However, the mechanism remains unclear. As in Zhang et al.^11^, we implemented a transient decrease of the phosphorylation rate constant $k_{f,p38}$ by $[E_{5 - HT} ]$ (Eqs. 24–25). *[p38*^*pp*^*]* corresponds to the p-p38 MAPK level (*i.e.,* p38 MAPK activity) measured by immunofluorescence. The equations describing the p38 MAPK pathway are therefore,

16 $$\frac{{d[Raf_{p38}^{p} ]}}{dt} = (k_{basal,Rafp38} + k_{f,Rafp38} [5 - HT])[Raf_{p38} ] - k_{b,Rafp38} [Raf_{p38}^{p} ]$$

17 $$[Raf_{p38} ] = [Raf_{p38} ]_{total} - [Raf_{p38}^{p} ]$$

18 $$\frac{{d[MEK_{p38} ]}}{dt} = \frac{{k_{b,MEK} [MEK_{{^{p38} }}^{p} ]}}{{[MEK_{{^{p38} }}^{p} ] + K_{MEK,2} }} - \frac{{k_{f,MEK} [Raf_{{^{p38} }}^{p} ][MEK_{p38} ]}}{{[MEK_{p38} ] + K_{MEK,1} }}$$

19 $$\frac{{d[MEK_{{^{p38} }}^{pp} ]}}{dt} = \frac{{k_{f,MEK} [Raf_{{^{p38} }}^{p} ][MEK_{{^{p38} }}^{p} ]}}{{[MEK_{{^{p38} }}^{p} ] + K_{MEK,1} }} - \frac{{k_{b,MEK} [MEK_{{^{p38} }}^{pp} ]}}{{[MEK_{{^{p38} }}^{pp} ] + K_{MEK,2} }}$$

20 $$[MEK_{{^{p38} }}^{p} ] = [MEK_{p38} ]_{total} - [MEK_{p38} ] - [MEK_{{^{p38} }}^{pp} ]$$

21 $$\frac{d[P38]}{{dt}} = \frac{{k_{b,p38} [P38^{p} ]}}{{[P38^{p} ] + K_{p38,2} }} - \frac{{k_{f,p38} [P38]}}{{[P38] + K_{p38,1} }}$$

22 $$\frac{{d[p38^{pp} ]}}{dt} = \frac{{k_{f,p38} [p38^{p} ]}}{{[p38^{p} ] + K_{p38,1} }} - \frac{{k_{b,p38} [p38^{pp} ]}}{{[p38^{pp} ] + K_{p38,2} }} \,$$

23 $$[p38^{p} ] = [p38]_{total} - [p38] - [p38^{pp} ]$$

24 $$k_{f,p38} = \frac{{k_{f,p38\_RSK} [RSK^{p} ] + k_{f,p38\_MEK} [MEK_{{^{p38} }}^{pp} ]}}{{1 + [E_{5 - HT} ]}}$$

25 $$\frac{{d[E_{5 - HT} ]}}{dt} = k_{E5HT} \frac{[5 - HT]}{{[5 - HT] + K_{5HT\_p38} }} - k_{d,E5HT} [E_{5 - HT}]$$

#### RSK pathway

RSK is a new kinase added to the model. Considering the empirical results in this study and in Liu et al.^23^, we hypothesize that 5-HT engages two pathways to activate RSK. To simplify the model, the immediate transient activation of RSK by 5-HT is directly regulated by PKA (Fig. 3A, pathway 11), whereas the delayed activation of RSK is via the Raf/MEK/ERK pathway (Fig. 3A, pathway 4 → 5 → 6 → 7). *[RSK*^*p*^*]* corresponds to the pRSK level (*i.e.,* RSK activity) measured by immunofluorescence. The equations describing the RSK pathway are,

26 $$\begin{gathered} \frac{{d[RSK^{p} ]}}{dt} = \{ k_{PKAc,RSK} ([PKA_{C} ] - [PKA_{C} ]_{basal} )^{ + } ([PKA_{C} ] - [PKA_{C} ]_{basal} ) \hfill \\ \, + k_{ERK,RSK} [ERK^{pp} ]\} [RSK] - k_{b,RSK} \frac{{[RSK^{p} ]}}{{[RSK^{p} ] + K_{b,RSK} }} \hfill \\ \end{gathered}$$

27 $$[RSK] = [RSK]_{total} - [RSK^{p} ]$$

### Extension of the model to include the CREB1/2 and *Aplysia* tolloid/BMP-1-like (TBL) and TGF-β pathways

To build a more complete model of signal transduction pathways in sensory neurons, the model (Fig. 3A) was extended (Fig. 4) to include the transcriptional activator CREB1, the transcriptional repressor CREB2, and the *Aplysia* tolloid/BMP-1-like protein (TBL) and TGF-β (TGF-β) pathways. TGF-β signaling is a critical mechanism underlying LTF and is also believed to underlie the sustained activation of the MAPK pathway by LTM-inducing training protocols^13–15,17,42,52,60^. TGF-β is believed to be activated by release from sensory neurons of a tolloid/BMP-1-like peptide TBL, and in turn enhances the activation of ERK and CREB1^13–15,61,62^. *Tolloid/BMP-1* belongs to a developmentally regulated gene family. Both *tolloid* and *BMP-1* encode metalloproteases that activates TGF-β. The amount of mRNA for TBL in *Aplysia* sensory neurons increases after 5-HT treatment^61^. Expression of TBL is likely to be induced by CREB1^13–15,63,64^, suggesting an ERK—CREB1—TBL—TGF-β—ERK—CREB1 positive feedback loop (Fig. 4, pathway 7 → 16 → 19 → 21 → 22 → 23 → 7). Activation of the TGF-β cascade by two pulses of 5-HT is significantly increased after the second pulse compared to the first pulse^62^. When BMP-1 protein was combined with one pulse of 5-HT, TGF-β activity was also significantly enhanced. Thus, it appears that an increase of the BMP-1 like protein TBL can activate sufficient TGF-β to induce an ERK—TGF-β—ERK positive feedback loop, leading to a sustained increase of pERK.

#### CREB1/2 and TBL pathways

The differential equations describing the phosphorylation of CREB1/2 were adapted from Liu et al.^33^ that successfully predicted two different rescue protocols to restore LTF impaired by knock down of CREB binding protein (CBP) or of CREB1^33,65^. The changes from the previous model are the inclusion of two new pathways: the phosphorylation of CREB1 by RSK (Eq. 28) and the phosphorylation of CREB2 by p-p38 MAPK (Eq. 31).

After 5-HT treatment, RSK and PKA activate (i.e., phosphorylate) CREB1 (pCREB1) (Fig. 4, pathway 15–16) (Eq. 28), p38 MAPK activates CREB2 (Fig. 4, pathway 18) (Eq. 31), and ERK inactivates CREB2 (Fig. 4, pathway 17) (Eq. 30). CREB1 and CREB2 both bind to DNA at sequences termed cAMP response elements. We hypothesized that active CREB1 and CREB2 respectively induce and repress TBL expression (Fig. 4, pathway 19—20). [TBL] denotes the level of TBL (Eq. 33).

The equations describing dynamics of CREB1, CREB2, and TBL are,

28 $$\begin{gathered} \frac{d[pCREB1]}{{dt}} = \{ k_{RSK,CREB1} ([RSK^{p} ] - [RSK^{p} ]_{basal} )^{ + } ([RSK^{p} ] - [RSK^{p} ]_{basal} ) \hfill \\ \, + k_{PKA,CREB1} ([PKA_{C} ] - [PKA_{C} ]_{basal} )^{ + } ([PKA_{C} ] - [PKA_{C} ]_{basal} )\} [CREB1] \, \hfill \\ \, - k_{pphos1} [pCREB1] \hfill \\ \end{gathered}$$

29 $$[CREB1]_{unphos} = [CREB1]_{total} - [pCREB1]$$

30 $$\frac{{d[pCREB2_{{^{ERK} }} ]}}{dt} = k_{ERK,CREB2} [ERK^{PP} ][CREB2]_{unphos} - k_{pphos2} [pCREB2_{{^{ERK} }} ]$$

31 $$\frac{{d[pCREB2_{{^{p38} }} ]}}{dt} = k_{P38,CREB2} [p38^{PP} ][CREB2]_{unphos} - k_{pphos2} [pCREB2_{{^{p38} }} ]$$

32 $$[CREB2]_{unphos} = [CREB2]_{total} - [pCREB2_{{^{p38} }} ] - [pCREB2_{{^{ERK} }} ]$$

33 $$\frac{d[TBL]}{{dt}} = \frac{{\frac{{[pCREB1]^{2} }}{{K_{CREB1,TGF}^{2} }}}}{{1 + \frac{{[pCREB1]^{2} }}{{K_{CREB1,TGF}^{2} }} + \frac{{[CREB2]_{unphos}^{2} }}{{K_{CREB2unphos,TGF}^{2} }} + \frac{{[pCREB2_{{^{p38} }} ]^{2} }}{{K_{CREB2P38,TGF}^{2} }}}} - [TBL]$$

Most parameter values of the CREB1/2 equations are as described previously (Liu et al.)^33^ (Table S2). Because RSK was added to phosphorylate CREB1, phosphorylation rate constants of CREB1 (*k*_*RSK,CREB1*_, *k*_*PKA,CREB1*_) were adjusted so that the concentration of pCREB1 in the extended model remained similar to that simulated in Liu et al.^33^. The parameter values of the equations describing the phosphorylation of CREB2 by pERK are the same as in Liu et al.^33^, and the same set of parameter values was used in the equations describing the phosphorylation of CREB2 by p-p38 MAPK.

The equation for TBL was adapted from the equation for C/EBP in Liu et al.^33^, but the effect of *pCREB2*_*p38*_ was added. It is not clear from data whether unphosphorylated CREB2 is a transcription repressor^29,30,51^. We assumed that both CREB2 activated by p38 MAPK (*pCREB2*_*p38*_ in Eq. 31) and unphosphorylated CREB2 (*pCREB2*_*unphos*_ in Eq. 30) will suppress the expression of TBL, but the effect of *pCREB2*_*p38*_ may be predominant^29,30^. Therefore, the constant *K*_*CREB2P38,TGF*_ in Eq. 33 is 10 times smaller than *K*_*CREB2unphos,TGF*_ (i.e., more sensitive).

#### Addition of the TGF-β pathway to close an extracellular feedback loop

The TGF-β pathway (Fig. 4, pathway 21 → 22 → 23) was added to complete the extracellular feedback loop necessary to sustain levels of pERK. The equations describing the regulation of TBL and TGF-β by CREB1/CREB2 are more speculative than those for the CREB1/2 and TBL pathways described above, but do simulate the salient features of their dynamics. We assumed that TGF-β activity can be indirectly (via TBL expression) enhanced by CREB1 and repressed by CREB2 (Fig. 4, pathway 19 → 21; 20 → 21) and increased TGF-β in turn activates ERK (Fig. 4, pathway 22 → 23), forming a positive feedback loop (termed ‘ERK—TGF-β feedback loop’) (Fig. 4, pathway 7 → 16 → 19 → 21 → 22 → 23 → 7). The dynamics of TGF-β after two pulses of 5-HT are unclear. However, the characteristic feature of a positive feedback loop is to enhance the nonlinearity of a network and, if of sufficient strength, to increase pERK from a lower state to a higher state for hours or longer (i.e., the existence of two steady states for pERK)^66, 67^.

Equations describing the activation of TGF-β by TBL (Eq. 34) and activation of ERK by TGF-β (Eqs. 35–40) were as follows. [TGF-β] denotes the level of activated TGF-β.

34 $$\frac{d[TGF - \beta ]}{{dt}} = k_{ApTBL,TGF} \frac{{[TBL]^{2} }}{{[TBL]^{2} + K_{ApTBL,TGF}^{2} }} - k_{d,TGF} [TGF - \beta ]$$

To simplify the model, we assumed that Trk and TGF-β induce similar, but distinct pathways to phosphorylate MEK in different subcellular locations, which converge to phosphorylate ERK in the soma, a plausible hypothesis suggested by Kopec et al.^17^. To implement this hypothesis, and to minimize the number of parameters, the model incorporates a new variable *MEK*_*TGF*_ to denote MEK activated by the pathway regulated by TGF-β in Eqs. (35–38), to distinguish from MEK activated by the NT pathway (*MEK*_*TrkB*_) in Eqs. (3–5). We assumed p38 MAPK has the same effects on these two MEK activation pathways (pathways 9 and 24).

35 $$\frac{{d[MEK_{TGF} ]}}{dt} = \frac{{k_{{b,MEK_{TGF} }} [MEK_{TGF}^{p} ]}}{{[MEK_{{_{TGF} }}^{p} ] + K_{MEK,2} }} - \frac{{k_{f,MEK} [TGF - \beta ][MEK_{TGF} ]}}{{[MEK_{{_{TGF} }} ] + K_{MEK,1} }}$$

36 $$\frac{{d[MEK_{TGF}^{pp} ]}}{dt} = \frac{{k_{f,MEK} [TGF - \beta ][MEK_{TGF}^{p} ]}}{{[MEK_{TGF}^{p} ] + K_{MEK,1} }} - \frac{{k_{{b,MEK_{TGF} }} [MEK_{TGF}^{pp} ]}}{{[MEK_{TGF}^{pp} ] + K_{MEK,2} }}$$

37 $$[MEK_{TGF}^{p} ] = [MEK_{TGF} ]_{total} - [MEK_{TGF} ] - [MEK_{TGF}^{pp} ]$$

38 $$k_{{b,MEK_{TGF} }} = k_{b,MEK\_basal} + k_{b,MEK\_p38} ([MEK_{TGF}^{pp} ] - [MEK_{TGF}^{pp} ]_{basal} )^{ + }[ E_{p38 - MEK}]$$

Equations describing pERK dynamics (Eqs. 6–7) were modified to include the regulation of TGF-β

39 $$\frac{d[ERK]}{{dt}} = \frac{{k_{b,ERK} [ERK^{p} ]}}{{[ERK^{p} ] + K_{ERK,2} }} - \frac{{k_{f,ERK} ([MEK_{TrkB}^{pp} ] + [MEK_{TGF}^{pp} ])[ERK]}}{{[ERK] + K_{ERK,1} }}$$

40 $$\frac{{d[ERK^{pp} ]}}{dt} = \frac{{k_{f,ERK} ([MEK_{TrkB}^{pp} ] + [MEK_{TGF}^{pp} ])[ERK^{p} ]}}{{[ERK^{p} ] + K_{ERK,1} }} - \frac{{k_{b,ERK} [ERK^{pp} ]}}{{[ERK^{pp} ] + K_{ERK,2} }} \,$$

The parameter values governing regulation of MEK_TGF_ by TGF-β are adapted from those governing regulation of MEK_TrkB_ by the NT-Trk pathway (Table S2). Our preliminary empirical results suggest that the sustained increase of pERK after two pulses of 5-HT lasts ~ 3 h, thus *k*_*ApTBL,TGF*_ = 0.0087 μM/min, *k*_*d,TGF*_ = 0.0058 min^−1^ so that TBL induced by two pulses of 5-HT can activate sufficient TGF-β and the sustained increase of pERK declines at ~ 3 h (Fig. 5).

#### Adjustment of parameters

Standard parameter values in this study were adapted from previous studies^11,28,33,55^, the majority of which were unchanged. However, modifications of some parameter values were necessary, and new parameters were added, due to addition of new pathways or revision of existing pathways. All model parameter values are given in Table S2. The process of parameter adjustment was as follows:

The parameters were first adjusted by trial and error to fit the dynamics of pERK, pRSK, p-p38 MAPK and PKAc after one pulse of 5-HT. The dynamics of pERK and p-p38 MAPK are from Zhang et al.^11^. The dynamics of pRSK and PKAc are from Figs. 1, 2. The parameters of the equation describing *NT* were constrained so that the NT/ Trk pathway remains, at least partially, activated for 1 h after one pulse of 5-HT (green time course in Fig. 5A7). These constraints are based on empirical findings that: (1) Inhibiting Trk for ~ 1 h suppressed the increase of MAPK activity at 45 min after one stimulus^17^, suggesting that delayed MAPK activation at 45 min is due to Trk activity. (2) Applying the p38 MAPK inhibitor SB203580 for 1 h after one pulse of 5-HT prevented the return of pERK to the basal level at 60 min after 5-HT^11^, suggesting that the decrease of pERK from 45 to 60 min post-onset of 5-HT is due to p38 MAPK-mediated late inhibition of the MEK/ERK pathway (Fig. 3A, pathway 9), which opposes the activating effects of the Trk pathway. The initial set of parameter values was then fine-adjusted in a range of 5% ~ 20%, by trial and error, to obtain a better fit between empirical and simulation results of kinase dynamics after one pulse. Finally, the corresponding pathways in Fig. 3A were blocked to simulate the effects of PKA or RSK inhibitors used in Fig. 2, to confirm that the final set of parameter values simulates the empirical results of Fig. 2.

#### Numerical methods

Fourth-order Runge–Kutta integration was used for integration of all differential equations with a time step of 3 s. Further time step reduction did not lead to significant improvement in accuracy. The steady-state levels of variables were determined after at least one simulated day, prior to any manipulations. The model was programmed in XPPAUT (http://www.math.pitt.edu/~bard/xpp/xpp.html). ^68^ and simulated on Dell Precision T1700 microcomputers. Source codes will be submitted to the ModelDB database^69^, and to GitHub (https://github.com).

## Supplementary Information

Supplementary Information.

## Data Availability

All data from empirical studies are available upon request. Source codes of the computational model will be submitted to the ModelDB database^61^, and to GitHub (https://github.com).
