# Supplementary material for: Quantitative description of the interactions among kinase cascades underlying long-term plasticity of Aplysia sensory neurons
Source: Sci Rep. 2021 Jul 22;11:14931. doi: 10.1038/s41598-021-94393-0 (PMC8298407; doi:10.1038/s41598-021-94393-0)
Supplement: Supplementary file 1 — Supplementary Information. [file 41598_2021_94393_MOESM1_ESM.docx]

Title: Quantitative description of the interactions among kinase cascades underlying long-term plasticity of *Aplysia* sensory neurons

**Authors:** Yili Zhang^1^, Paul D. Smolen^1^, Leonard J. Cleary^1^, and John H. Byrne^1^*

**Affiliations:**

^1^ Department of Neurobiology and Anatomy

W.M. Keck Center for the Neurobiology of Learning and Memory

McGovern Medical School

The University of Texas Health Center at Houston;

6431 Fannin Street, Suite MSB 7.046

Houston, TX 77030.

*Corresponding author. John H. Byrne, Ph.D., Department of Neurobiology and Anatomy, W.M. Keck Center for the Neurobiology of Learning and Memory, McGovern Medical School, The University of Texas Health Center at Houston, TX, Houston, TX 77030. Tel: 713-500-5602, Fax: 713-500-0623. E-mail: [John.H.Byrne@uth.tmc.edu](mailto:John.H.Byrne@uth.tmc.edu)

**Supplementary Figures and Tables**

**
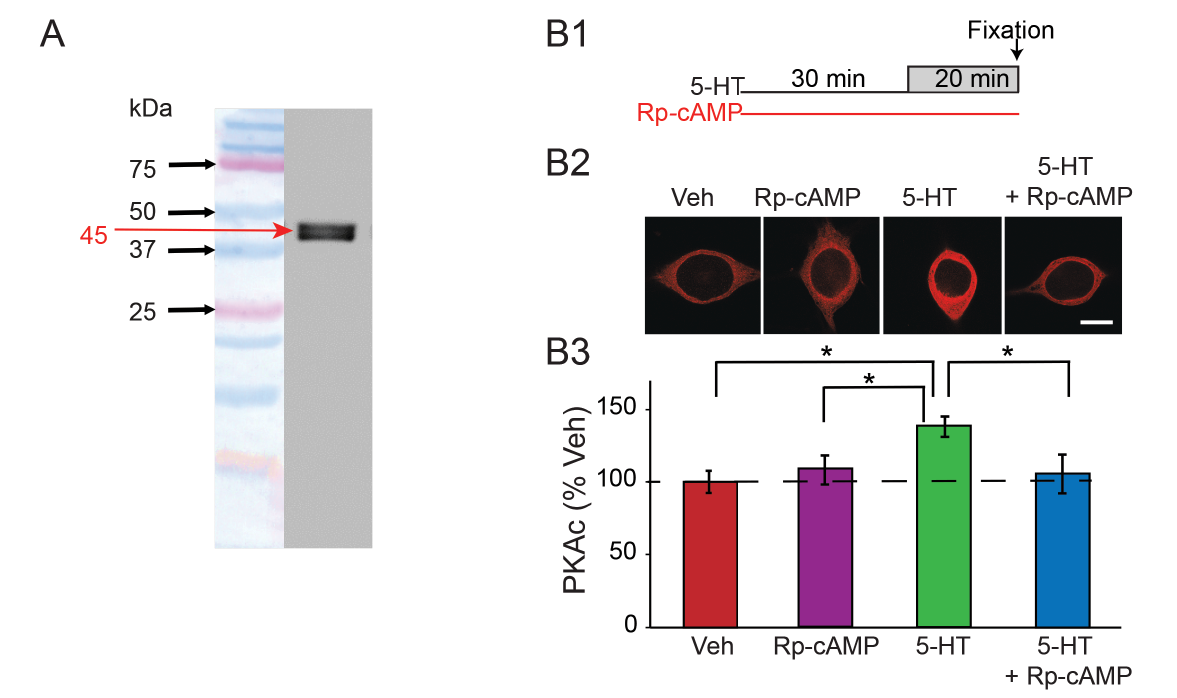
**

**Figure S1**. Anti-PKAc antibody validation. **A**, Western blot of PKAc. Whole cell extracts of *Aplysia* nervous system were used for Western blot analyses^23,33^. The antibody recognized a single band with a molecular weight of ~45 kDa, consistent with the expected size of *Aplysia* PKAc^34^. **B1**, Protocol for applying 5-HT with the PKA inhibitor Rp-cAMP. Rp-cAMP (10 μM, Calbiochem) was applied to SN cultures for 50 min starting 30 min before 5-HT. **B2**, Representative confocal images of PKAc after 20 min 5-HT, in the absence or presence of Rp-cAMP. **B3**, Summary data. 5-HT alone significantly increased immunofluorescence by 38.3 ± 6.5% (n = 9). Antibody binding was thus reduced to a level comparable to the control (4.6 ± 11.4%, n = 9) (statistical analyses in Table S1). These data suggest that increase of PKAc was induced by 5-HT via the cAMP pathway. Data are represented as mean ± SEM. All scale bars are 40 μm. * p< 0.05.

**
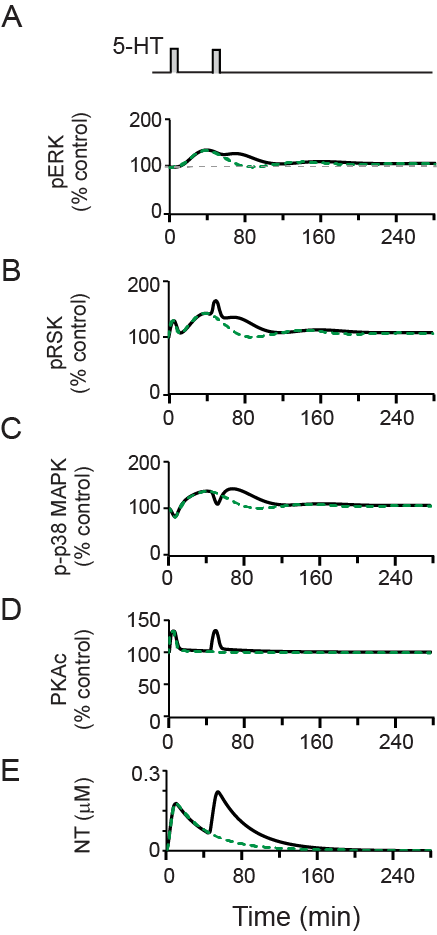
**

**Figure S2**. Simulated dynamics of pERK, pRSK, p-p38 MAPK, PKAc and NT, after one or two 5-min pulses of 5-HT with ISI of 45 min based on model of Fig. 3A. Black curves are simulations after two pulses of 5-HT. Green curves are simulations after one pulse of 5-HT.

**
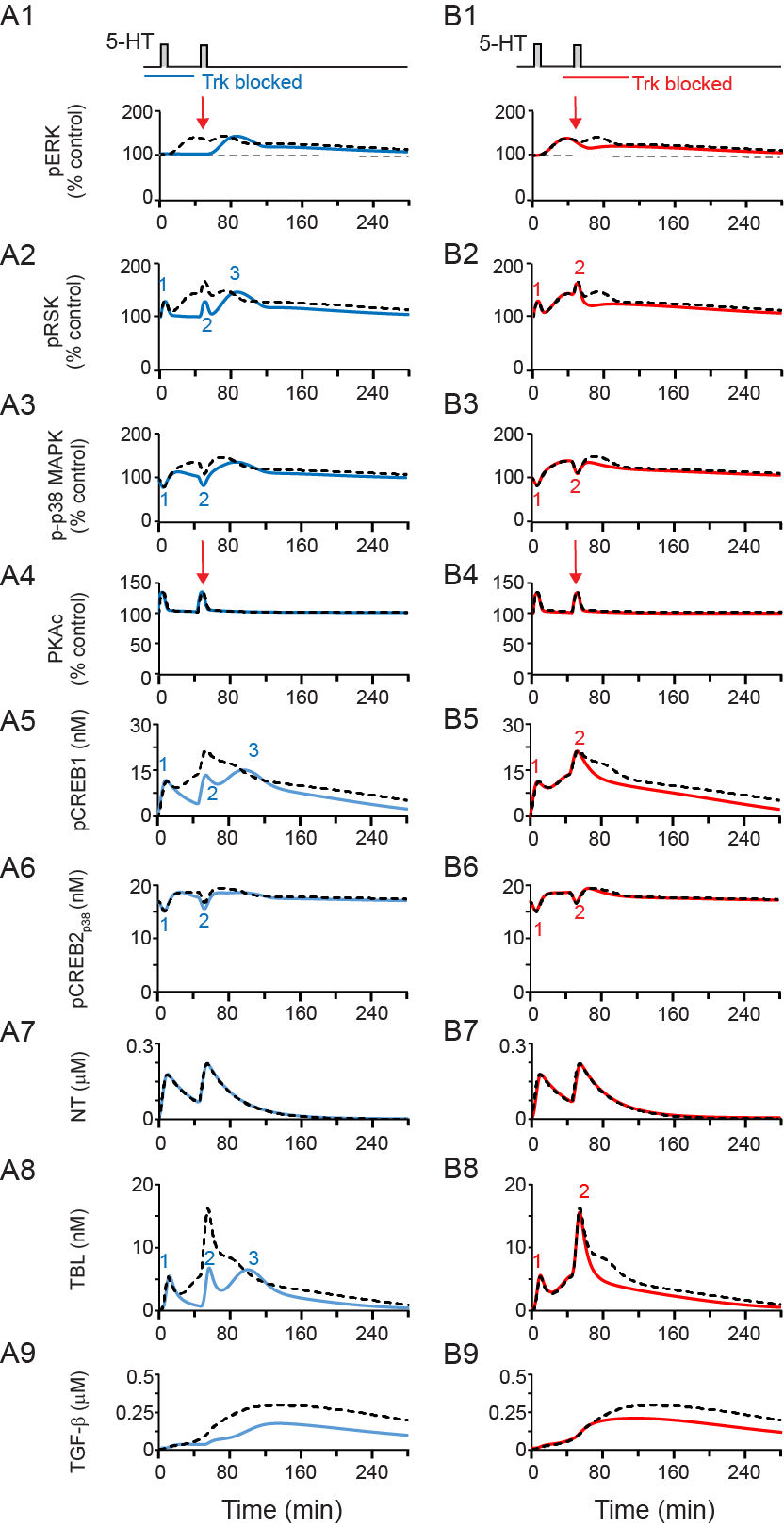
**

**Figure S3**. Simulated dynamics of pERK, pRSK, p-p38 MAPK, PKAc, pCREB1, pCREB2_p38_, NT, TBL and TGF-β levels after two 5-min pulses of 5-HT with ISI of 45 min, with block of Trk applied during the first (A) or second (B) pulse of 5-HT. To investigate whether the NT/Trk-ERK pathway activated by the first pulse of 5-HT is necessary for activating the ERK - TGF-β feedback loop, we simulated the application of a Trk inhibitor during the first pulse of 5-HT. [NT] in Eq. 1 of the model was set to zero from 10 min prior to the first pulse to 5 min prior to the second pulse (A)^17^. Blue and red curves are simulations with inhibitors, black dashed curves are control simulations without inhibitors. Compared to control without Trk inhibition, the pERK increase was delayed until after the second pulse, but then remained elevated for hours, close to control (A1). Grey dashed line represents 100% control. We next repeated the simulation except NT was instead set to zero from 5 min prior to 1 h post-onset of the second pulse (B)^17^. Compared to control, only one wave of increase in pERK was induced by the NT-ERK pathway with Trk blocked (B1). However, at later time points (> 1 h after the second pulse), pERK levels were persistently elevated. Both simulations replicated the data of Kopec et al. (2015)^17^, suggesting activation of Trk – ERK during either the first or second pulse is not necessary to activate the ERK - TGF-β feedback loop. Instead, NT/Trk – ERK independent pathways (pathway 1→11, 5-HT- PKA - RSK, and pathway 10, 5-HT - p38 MAPK, Figs. 3A&4) produce overlapped increases of PKAc and pERK (red arrows), which contribute to the activation of RSK, regulation of CREB1/2 and TBL, and induction of TGF-β, when the Trk-ERK pathway is temporarily blocked. Numbers “1”, “2”, and “3” in A2, A5, A8, B2, B5, and B8 represent waves of increase. Numbers “1” and “2” in A3, A6, B3, and B6 represent waves of decrease.

**
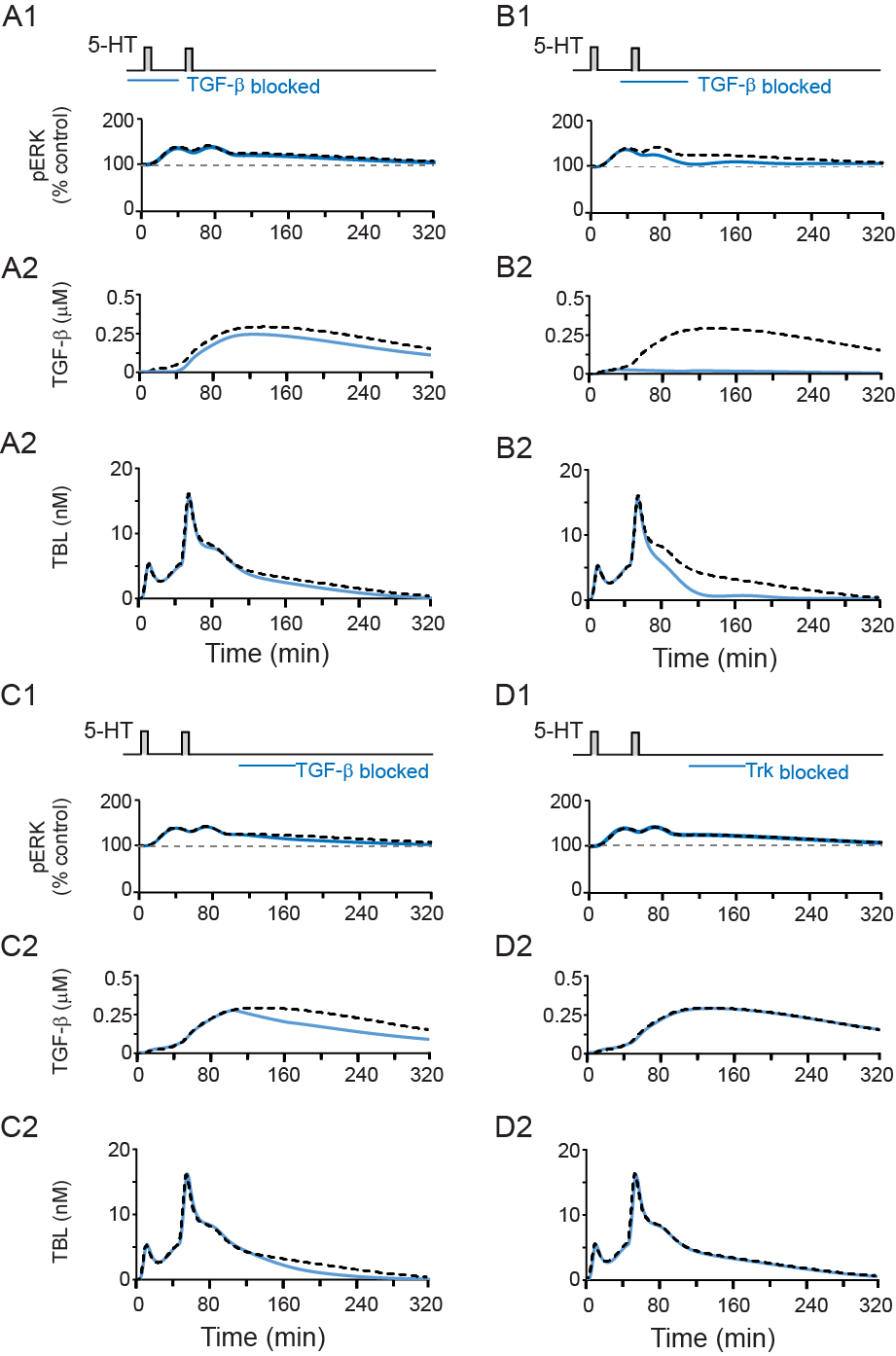
**

**Figure S4**. Simulated dynamics of pERK, TGF-β and TBL levels after two 5-min pulses of 5-HT with ISI of 45 min, with block of TGF-β applied during the first (A), or second (B) pulse of 5-HT, or 1 h after 5-HT (C), or with block of Trk applied 1 h after 5-HT (D). Blue curves are simulations with inhibitors, black dashed curves are control simulations without inhibitors.

| **Fig.2A one-way RM ANOVA (F_3,27_ = 6.275, *P* = 0.002), pairwise comparisons (Student-Newman-Keuls)** | | | |
| --- | --- | --- | --- |
|  | q | P | n |
| 5‑HT alone vs. Veh group | 5.642 | 0.003 | 10 |
| 5‑HT alone vs. KT5720 alone group | 4.039 | 0.008 | 10 |
| 5‑HT alone vs. 5-HT + KT5720 group | 4.827 | 0.006 | 10 |
| KT5720 alone vs. Veh group | 1.603 | 0.502 | 10 |
| 5-HT + KT5720 vs. Veh group | 0.815 | 0.569 | 10 |
| KT5720 alone vs. 5-HT + KT5720 group | 0.788 | 0.582 | 10 |
| **Fig.2B one-way RM ANOVA (F_3,15_ = 5.943, *P* = 0.007),** **pairwise comparisons (Student-Newman-Keuls)** | | | |
|  | q | P | n |
| 5‑HT alone vs. Veh group | 5.368 | 0.009 | 6 |
| 5‑HT alone vs. Rp-cAMP alone group | 4.292 | 0.009 | 6 |
| 5‑HT alone vs. 5-HT + Rp-cAMP group | 4.725 | 0.012 | 6 |
| Rp-cAMP alone vs. Veh group | 1.076 | 0.732 | 6 |
| 5-HT + Rp-cAMP vs. Veh group | 0.643 | 0.656 | 6 |
| Rp-cAMP alone vs. 5-HT + Rp-cAMP group | 0.433 | 0.764 | 6 |
| **Fig.2C one-way RM ANOVA (F_3,18_ = 4.793, *P* = 0.013),** **pairwise comparisons (Student-Newman-Keuls)** | | | |
|  | q | P | n |
| 5‑HT alone vs. Veh group | 3.751 | 0.041 | 7 |
| 5‑HT alone vs. KT5720 alone group | 5.189 | 0.009 | 7 |
| 5‑HT alone vs. 5-HT + KT5720 group | 3.159 | 0.039 | 7 |
| KT5720 alone vs. Veh group | 1.438 | 0.323 | 7 |
| 5-HT + KT5720 vs. Veh group | 0.592 | 0.681 | 7 |
| KT5720 alone vs. 5-HT + KT5720 group | 2.030 | 0.345 | 7 |
| **Fig.2D one-way RM ANOVA (F_3,24_ = 3.457, *P* = 0.032),** **pairwise comparisons (Student-Newman-Keuls)** | | | |
|  | q | P | n |
| 5‑HT alone vs. Veh group | 4.183 | 0.033 | 9 |
| 5‑HT alone vs. Rp-cAMP alone group | 3.561 | 0.048 | 9 |
| 5‑HT alone vs. 5-HT + Rp-cAMP group | 3.077 | 0.040 | 9 |
| Rp-cAMP alone vs. Veh group | 0.622 | 0.664 | 9 |
| 5-HT + Rp-cAMP vs. Veh group | 1.106 | 0.718 | 9 |
| Rp-cAMP alone vs. 5-HT + Rp-cAMP group | 0.484 | 0.736 | 9 |
| **Fig.2E one-way RM ANOVA (F_3,15_ = 15.437, *P* <0.001),** **pairwise comparisons (Student-Newman-Keuls)** | | | |
|  | q | P | n |
| 5‑HT alone vs. Veh group | 8.303 | 0.001 | 6 |
| 5‑HT alone vs. U0126 alone group | 5.92 | 0.002 | 6 |
| 5‑HT alone vs. 5-HT + U0126 group | 1.134 | 0.435 | 6 |
| U0126 alone vs. Veh group | 2.384 | 0.113 | 6 |
| 5-HT + U0126 vs. Veh group | 7.169 | 0.001 | 6 |
| 5-HT + U0126 vs. U0126 alone group | 4.785 | 0.004 | 6 |
| **Fig. 2F Friedman repeated measures analysis of variance on ranks (**χ**-square = 13.93, 3 d.o.f, *P* = 0.003), pairwise comparisons (Student-Newman-Keuls)** | | | |
|  | q | P | n |
| 5-HT alone vs. Veh group | 4.906 | < 0.05 | 9 |
| 5-HT alone vs. BI-D1870 alone group | 5.333 | < 0.05 | 9 |
| 5-HT alone vs. 5-HT + BI-D1870 group | 5.185 | < 0.05 | 9 |
| BI-D1870 alone vs. Veh group | 1.414 | > 0.05 | 9 |
| 5-HT + BI-D1870 vs. Veh group | 2.667 | > 0.05 | 9 |
| BI-D1870 alone vs. 5-HT + BI-D1870 group | 2.357 | > 0.05 | 9 |
| **Fig. 5C one-way RM ANOVA (F_3,18_ = 5.758, *P* = 0.006),** **pairwise comparisons (Student-Newman-Keuls)** | | | |
| 5-HT alone vs. Veh group | 5.373 | 0.007 | 7 |
| 5-HT alone vs. TrkB Fc alone group | 4.587 | 0.012 | 7 |
| 5-HT alone vs. 5-HT + TrkB Fc group | 4.077 | 0.010 | 7 |
| TrkB Fc alone vs. Veh group | 0.786 | 0.585 | 7 |
| 5-HT + TrkB Fc vs. Veh group | 1.297 | 0.637 | 7 |
| TrkB Fc alone vs. 5-HT + TrkB Fc group | 0.510 | 0.722 | 7 |
| **Fig. S1 one-way RM ANOVA (F_3,24_ = 4.762, *P* = 0.010), pairwise comparisons (Student-Newman-Keuls, SNK)** | | | |
|  | q | P | n |
| 5‑HT alone vs. Veh group | 4.801 | 0.012 | 9 |
| 5‑HT alone vs. Rp-cAMP alone group | 3.752 | 0.014 | 9 |
| 5‑HT alone vs. 5-HT + Rp-cAMP group | 4.284 | 0.015 | 9 |
| Rp-cAMP alone vs. Veh group | 1.049 | 0.741 | 9 |
| 5-HT + Rp-cAMP vs. Veh group | 0.517 | 0.718 | 9 |
| Rp-cAMP alone vs. 5-HT + Rp-cAMP group | 0.532 | 0.710 | 9 |

**Table S1.** **Statistical analysis of pairwise comparisons.**

| Parameter values unchanged from the original models:  *k_f,MEK_* = 0.41 min^−1^, *k_b,MEK_basal_* = 0.04 μM/min, *k_b,MEK_p38_* = 0.04 min^−1^, *K_MEK,1_* = 0.20 μM, *K_MEK,2_* = 0.19 μM, *k_f,ERK_* = 0.41 min^−1^, *k_b,ERK_* = 0.12 μM/min, *K_ERK,1_* = 0.19 μM, *K_ERK,2_* = 0.21 μM, *[ERK]_tota_*_l_ = 0.5 μM, *k_basal,Rafp38_* = 0.036 min^−1^, *k_b,Rafp38_*  = 0.1 min^−1^, *[Raf_p38_]_total_* = 0.5 μM, *[MEK _p38_]_total_* = 0.5 μM, *k_b,p38_* = 0.12 μM/min, *K_p38,1_* = 0.19 μM, *K_p38,2_* = 0.21 μM, *[p38]_tota_*_l_ = 0.5 μM, *K_5HT_p38_* = 50 μM, λ = 3.64 μM/min, *K_5HT_* = 85 μM, *k_b,cAMP_* = 1 min^−1^, *k_f,PKA_* = 20 μM^−2^min^−1^*,* *k_b,PKA_* = 12 μM^−1^min^−1^, *k_pphos1_* = 0.05 min^−1^, *[CREB1]_total_* = 0.05 μM, *k_ERK,CREB2_* = 3.5 μM^−1^min^−1^, *k_pphos2_* = 0.5 min^−1^, *[CREB2]_total_* = 0.05 μM |
| --- |
| Parameter values changed from the original models:  *k_f,Raf_* = 0.037 μM^−1^min^−1^, *k_basal,Raf_* = 0.00013 min^−1^, *k_b,Raf_*  = 0.00038 min^−1^,  *k_EP38,MEK_* = 0.33 μM^−1^ min^−1^, *k_d,EP38,MEK_* = 0.0013 min^−1^, *k_f,Rafp38_* = 0.0009 μM^−1^min^−1^, *k_E5HT_* = 0.8 min^−1^, *k_d,E5_HT_* = 1 min^−1^, *k_PKA,CREB1_* = 0.3 μM^−1^min^−1^ |
| Values of new parameters:  *[Raf_TrkB_]_total_* = 0.5 μM, *[MEK_TrkB_]_total_* = 0.5 μM, *[MEK_TGF_]_total_* = 0.083 μM, *[ERK^pp^]_basal_* = 0. 14 μM,  *cAMP_bas_* = 0.7 μM, *[P38^pp^]_basal_* = 0.15 μM, *[MEK_TrkB_^pp^]_basal_* = 0.25 μM, *[MEK_TGF_^pp^]_basal_* = 0 μM, *k_f,p38_RSK_* = 0.99 min^−1^, *k_f,p38_MEK_* = 0.25 min^−1^, *K_TrkB_* = 12 μM, *k_f,NT_* = 0.3 μM/min, *[PKA_C_]_basal_* = 0.35μM, *K_PKAc,NT_* = 1 μM, *k_b,NT_* = 0.05 min^−1^,  *K_b,RSK_* = 0.2 μM, *[RSK]_tota_*_l_ = 0.5 μM, *[RSK^p^]_basal_* = 0.042 μM, *k_PKAc,RSK_* = 0.26 μM^−1^min^−1^*, k_ERK,RSK_* = 0.82 μM^−1^min^−1^, *k_b,RSK_* = 0.31 μM^−1^ min^−1^, *k_RSK,CREB1_* = 1.25 μM^−1^min^−1^, *k_P38,CREB2_* = 3.5 μM^−1^min^−1^, *k_f,ApTBL_* = 1 μM/min, *k_b,ApTBL_* = 1 min^−1^, *K_CREB1,TGF_* = 0.003 μM, *K_CREB2unphos,TGF_* = 0.003 μM, *K_CREB2P38,TGF_* = 0.0003 μM, *K_ApTBL,TGF_* = 0.0078 μM. |

**Table S2.** Parameter values of the model. Standard parameter values were adapted from our previous studies^11,28,33,56,66^. The majority were unchanged. However, changes in some values were necessary. Also, new parameters were added due to addition of new pathways or revision of existing pathways.
